# Selective EV Protein Sorting and Pathway Perturbation in AML Upon Synergistic FLT3 and Hedgehog Pathway Inhibition

**DOI:** 10.1002/jev2.70163

**Published:** 2025-09-23

**Authors:** Constantin Blöchl, Gabriele Blümel, Martin Wolf, Christof Regl, Heide‐Marie Binder, Suzana Tesanovic, Daniel Lankes, Nicole Maeding, Peter W. Krenn, Dirk Strunk, Fritz Aberger, Christian G. Huber

**Affiliations:** ^1^ Department of Biosciences and Medical Biology University of Salzburg Salzburg Austria; ^2^ Cell Therapy Institute Paracelsus Medical University Salzburg Austria; ^3^ Austian Red Cross Research GmbH OeRK Vienna Austria; ^4^ Cancer Cluster Salzburg Salzburg Austria; ^5^ Center for Tumor Biology and Immunology University of Salzburg Salzburg Austria

**Keywords:** AML, crenolanib, ErbB signalling, EVs, Hedgehog, HPI‐1, proteomics, ribosomal proteins

## Abstract

Acute myeloid leukaemia (AML) is a haematologic malignancy with high relapse incidence and mortality. Approximately one‐third of AML patients carry an fms‐like tyrosine kinase 3 (FLT3) mutation, often associated with GLI expression and Hedgehog signalling. AML cells shape their microenvironment into a leukaemia‐permissive space by releasing extracellular vesicles (EVs). EVs can transfer chemoresistance and thereby play an important role in refractory and relapsing diseases. Here, we discovered a synergistic effect of combined treatment with the FLT3 inhibitor Crenolanib and the Hedgehog pathway inhibitor HPI‐1 in the AML cell lines MOLM‐14 and MV4‐11. In‐depth comparative proteomics revealed alterations in the cellular and the EV proteome upon single or combined inhibition of FLT3 and GLI, highlighting affected pathways. By comparing cellular and EV proteomes, we found that transport of ribosomal proteins, such as RPS26 and RPL27A, and ErbB pathway members such as GAB1, GRB2 and SHC1 to EVs, is selectively avoided upon treatment with Crenolanib. These findings were corroborated by comparative proteomics of EVs derived from AML patients and healthy donors. Ribosomal and ErbB signalling pathway proteins may play an important role in microenvironmental modulation by EVs, and Crenolanib treatment potentially acts by interfering with leukaemia niche formation.

## Graphical abstract

We discovered synergistic effects of combined treatment of AML cell lines with FLT3 inhibitor Crenolanib and Hedgehog pathway inhibitor HPI‐1. Comparative proteomics revealed alterations between cellular and EV proteomes upon single or combined treatment, showing that C‐renolanib treatment selectively downregulates transport of ribosomal proteins and ErbB pathway members to EVs.

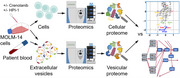

**Abbreviations**
AML: acute myeloid leukaemia
BET domain: bromodomain and extra‐terminal domain
CM: conditioned medium
Creno: Crenolanib
ECM: extracellular matrix
EVs: extracellular vesicles
FGSEA: fast gene set enrichment analysis
FLT3: FMS‐like tyrosine kinase 3
Glas: Glasdegib
GLI proteins: glioma‐associated oncogene transcription factors
Hh: Hedgehog
HPLC: high‐performance liquid chromatography
ITD: internal tandem duplication
MS: mass spectrometry
NES: normalized enrichment score
NK cell: natural killer cell
OXPHOS: oxidative phosphorylation
PCA: principal component analysis
PI3K: phosphoinositide 3‐kinase
SEC: size‐exclusion chromatography
SMO: Smoothened
TFF: tangential flow filtration
TRPS: tunable resistance pulse sensing
ZIP: zero interaction potency

## 1 Introduction

Acute myeloid leukaemia (AML) is the most common leukaemia in adults and is characterized by the malignant differentiation and uncontrolled proliferation of hematopoietic stem cells of the bone marrow followed by an infiltration of the peripheral blood and the spleen (Döhner et al. 2015). Despite new therapeutic approaches, AML remains a disease with high mortality, particularly due to the high rates of relapse and refractory disease, especially for those above the age of 60 (Döhner et al. 2015). Mutations in the FMS‐like tyrosine kinase 3 (*FLT3*) gene are frequent genetic drivers in patients diagnosed with AML, occurring in approximately one third of newly diagnosed AML patients (Papaemmanuil et al. 2016; Daver et al. 2019). The most prevalent FLT3 mutation is the internal tandem duplication (ITD), which accounts for approximately 25% of all AML diagnoses and is associated with a high leukemic load and unfavourable prognosis (Daver et al. 2019). In AML patients with FLT3‐ITD mutations, GLI proteins, key mediators of the Hedgehog (Hh) signalling pathway, which is required for normal embryonic development and stemness but is often hijacked by cancer cells, are frequently overexpressed (Lim et al. 2015; Aberger et al. 2017; Krenn and Aberger 2023; Tesanovic et al. 2022). Both, AML cell lines and bone marrowderived AML patient cells harbouring a FLT3‐ITD mutation have been shown to present active Hh signalling contribution to sustained AML cell viability and proliferation in the presence of FLT3‐inhibitors (Lim et al. 2015). Furthermore, in FLT3‐ITD/SmoM2 transgenic mice, which exhibit constitutive canonical Hh signalling, combined Hh and FLT3 inhibition reduced tumour growth and prolonged mouse survival (Lim et al. 2015). Additionally, a non‐canonical activation of GLI via FLT3 and its downstream target phosphoinositide 3‐kinase (PI3K) has been described for AML cells in vivo, and only combined inhibition of all three pathways prolonged survival of FLT3‐ITD AML‐transplanted mice (Latuske et al. 2017). The critical role of Hh signalling in the pathogenesis of AML has led to successful clinical trials resulting in the approval of the Hh pathway inhibitor Glasdegib (Glas) in combination with low‐dose chemotherapy for the treatment of AML patients (Cortes et al. 2019).

AML patients are often affected by relapse due to resistant subclones that survive chemotherapy or arise during treatment and then expand through clonal evolution (Papaemmanuil et al. 2016; Daver et al. 2019; Nehrbas et al. 2020). Importantly, resistance is driven by both cell intrinsic factors, such as acquired mutations and dysregulated signalling or enzyme activity, and cell‐extrinsic factors, including microenvironmental influences (Krenn and Aberger 2023; Tesanovic et al. 2022; Krenn et al. 2022). Recent approaches suggest that AML cells shape the vascular and endosteal niche in the bone marrow into a leukaemia‐permissive microenvironment through the release of extracellular vesicles (EVs) (Nehrbas et al. 2020). EVs are phospholipid bilayer membrane‐coated particles secreted by all types of cells. They allow the exchange of RNA, lipids and protein cargo between cells and are important for cell–cell communication (Colombo et al. 2014; Maas et al. 2017; Poupardin et al. 2024). Cancer cells often hijack the EV system of intercellular communication and release larger amounts of EVs into the microenvironment compared to healthy cells (Ab Razak et al. 2019; Mendes et al. 2024). Vesicular protein content in blood serum of AML patients was reportedly 60‐fold increased in comparison to healthy controls (Szczepanski et al. 2011). In addition to the rather unspecific EV protein markers such as CD9, CD63 and CD81, which have been reported in AML cell lines (Binder et al. 2021; Kang et al. 2021), CD33, CD34 and CD117 confirmed the EV's origin from myeloid blasts in a patient‐derived EV setting (Szczepanski et al. 2011). EVs play an essential role in the development of the primary tumour as well as in the metastatic stage (Becker et al. 2016). The number of vesicles as well as the cargo of EVs released by AML cells changes during disease progression and with therapy (Mendes et al. 2024; Hong et al. 2014). Strikingly, EVs allow the transfer of chemoresistance from resistant to sensitive cells (Nehrbas et al. 2020; Bouvy et al. 2017). In addition to the direct delivery of chemoresistance mechanisms, EVs support AML cell survival to pharmacological treatment in different ways. AML‐derived EVs additionally have immunomodulatory functions, suppressing NK cell‐mediated lysis of leukaemia cells by expressing multiple inhibitory proteins that bind to the surface receptors of NK cells (Nehrbas et al. 2020; Binder et al. 2021; Hong et al. 2017). In co‐culture experiments, EVs released from AML cells have been shown to interact with stromal cells to induce the release of interleukin‐8, which protects AML cells from chemotherapy (Chen et al. 2019). EVs released from chemotherapy‐treated cells are often referred to as chemoEVs, and there is increasing evidence that chemoEVs may be relevant to tumour behaviour in terms of metastasis, cancer stemness, relapse and immune response (Nehrbas et al. 2020; Ab Razak et al. 2019). Therefore, it is of great interest to understand how the cargo of EVs adapts to chemotherapy, especially in the case of the strongly microenvironment‐dependent AML. This could lead to the discovery of novel biomarkers, provide mechanistic insights into chemoresistance, and thus potentially identify therapeutic targets.

In general, the vesicular proteome has been well studied and extensively reviewed in several cancer entities, however, we lack a precise understanding of the role of EVs in AML pathogenesis (Xue et al. 2021; Greening et al. 2017). Proteomic data from multiple cell lines including AML cell lines derived from different tissues revealed that the cellular proteomes of the different cell lines are more correlated to each other than the vesicular proteomes, suggesting a selective protein sorting to EVs Carvalho et al. 2020. However, since EV trafficking plays a key role in chemoresistance development and transfer, recent studies have raised interest in how EV release and content is altered by chemotherapy. Most models showed that cancer cells release a high amount of EVs into the microenvironment after treatment with phototherapy or chemotherapy (Ab Razak et al. 2019; Aubertin et al. 2016; Bandari et al. 2018; Lv et al. 2012), but some reported no or the opposite effect (Ab Razak et al. 2019). More recently, the proteomic cargo of EVs released from apoptotic cells treated with either radio‐ or chemotherapy has been investigated. Proteomic analysis of EVs released from apoptotic cells from lethally irradiated glioblastoma cells revealed higher levels of spliceosomal proteins, which subsequently led to therapy resistance and proliferation of surviving cells (Pavlyukov et al. 2018). In lung adenocarcinoma cells treated with cisplatin or staurosporine, EVs were found to carry aldehyde dehydrogenase 1 family, member A1 (ALDH1A1), which further activated NF‐κB signalling and resulted in increased metastasis and stemness potential promoting epithelial‐mesenchymal transition (He et al. 2024).

To better understand the role of EVs in AML pathogenesis, we performed comprehensive proteomics both of AML cells and AML‐derived EVs. First, we compared cellular metabolic activity and viability after single or combined treatment of AML cells with FLT3 and/or Hh pathway inhibitors. For FLT3 inhibition we used Crenolanib (Creno), a promising drug shown to significantly increase the response rate, complete remission rate and relapse free survival of AML patients when combined with intensive chemotherapy (Wang et al. 2024). For Hh pathway inhibition we used either the Smoothened (SMO) inhibitor Glas or the GLI and BET bromodomain inhibitor HPI‐1 (Hyman et al. 2009; Bagka et al. 2023). While Glas specifically inhibits the Hh pathway component Smoothened (SMO) and therefore canonical Hh signalling (Munchhof et al. 2012), HPI‐1 affects Hh activity downstream of SMO most likely by targeting the Hh transcription factors GLI1, GLI2, and/or GLI3 (Hyman et al. 2009). Recently, HPI‐1 was shown to regulate GLI transcription and thus Hh pathway activity through interaction with bromodomain and extra‐terminal domain protein members; therefore, GLI may not be the only target (Bagka et al. 2023). Nevertheless, given conflicting data on Glas, showing benefits in patients ineligible for standard chemotherapy but not in a larger, general AML cohort treated with Glas plus intensive or non‐intensive chemotherapy (Krenn and Aberger 2023; Tesanovic et al. 2022), HPI‐1 may offer a promising alternative.

Back‐to‐back, we aim at implementing an unbiased proteomics approach to characterize and compare the proteomes of AML cells and their respective EVs during single and combined treatment with Creno and/or HPI‐1. Next to identifying adaptations in cellular and vesicular pathways upon treatment, we want to identify proteins that are either selectively transported into EVs or excluded from vesicular transport. Throughout all levels of proteomic analysis, global pathway analysis of the regulated proteins is expected to visualize how cancer cells adapt to selective targeted therapy through remodelling of their cellular proteome or adaptation in their EV release. Identified changes in the EV proteome upon treatment were corroborated by comparative EV proteomics of high‐risk AML patients and healthy donors.

## 2 Materials and Methods

### 2.1 Reagents and Materials

Creno was obtained from Selleckchem (CP‐868596; Boston, MA, USA), HPI‐1 was purchased from Sigma‐Aldrich (Hh1542; Vienna, Austria), Glas was received from Selleckchem (S7160). Stock solutions of pathway inhibitors were prepared in dimethyl sulfoxide (DMSO; Sigma‐Aldrich). Methanol (MeOH, ≥ 99.9%) and acetonitriIe (ACN, ≥ 99.9%) were acquired from VWR International (Vienna, Austria). Triethylammonium bicarbonate buffer (TEAB; pH 8.5 ± 0.1, 1.0 mol/L), sodium dodecyl sulfate (SDS; ≥ 99.5%), tris(2‐carboxyethyl)phosphine‐hydrochloride (TCEP; ≥ 98.0%), iodoacetamide (IAA; ≥ 99.0%), formic acid (FA; 98%–100%) and trifluoroacetic acid (TFA; ≥ 99.0%) were all purchased from Sigma–Aldrich. Ortho‐phosphoric acid (phosphoric acid; 85%) was obtained from Merck (Darmstadt, Germany). Trypsin (sequencing grade modified, porcine) was purchased from Promega (Madison, WI, USA). If not differently stated, solutions were prepared with ultrapure water produced in‐house with a MilliQ Integral 3 instrument (Millipore; Billerica, MA, USA).

### 2.2 Pharmacological Inhibition

MOLM‐14 or MV4‐11 cells were plated in 96 well plates. Cells were treated with Creno, HPI‐1 and Glas at varying concentrations for different time points as indicated in the respective figure. One tenth volume of AlamarBlue solution (Biorad; Hercules, CA, USA) was added to each well and absorption was measured at 570 and at 600 nm with a plate reader (Tecan; Männedorf, Switzerland). Measured metabolic activity was normalized to the respective controls. For synergy analysis, data was additionally normalized to respective extinction values after 8 h of treatment. From metabolic activity of different treatment combinations, the zero interaction potency (ZIP) synergy was calculated using the R package ‘SynergyFinder Plus’ (Zheng et al. 2022). For viability and proliferation assessment, 2.0 × 10^5^ trypan‐blue negative MOLM‐14 cells were seeded in RPMI medium (R8758; Sigma‐Aldrich) supplemented with 10% FCS (F7524, Sigma‐Aldrich), L‐glutamine (G7513, Sigma‐Aldrich) and Penicillin/Streptomycin (P0781, Sigma‐Aldrich). Cells were treated with DMSO as control, Creno and/or HPI‐1 at varying concentrations as described in the respective figure legends. Cells were harvested after 48 h of treatment and washed in PBS. Cell pellets were resuspended in 100 µL staining mix consisting of 1.0 µL Annexin‐FITC (Immunotools, Friesoythe, Germany), 1:1000 eFluor450 viability dye (Thermo Fisher Scientific, Waltham, MA, USA), 1.0 × 10^4^  flow cytometry counting beads (BioLegend, San Diego, CA, USA) in annexin buffer. Following a 10 min incubation, cells were washed, and pellets resuspended for flow cytometry. Analysis was performed on a Cytoflex S (Beckman Coulter, Vienna, Austria).

### 2.3 EV Preparation and Purification

MOLM‐14 cells were expanded in RPMI medium (R5886; Sigma–Aldrich) supplemented with 10% FBS (S 0615 Biochrom, Millipore) and 1.0% L‐alanyl‐L‐glutamine dipeptide (Dipeptiven; 11051014 Fresenius Kabi, Graz, Austria). 7.5 × 10^5^ MOLM‐14 cells/mL were transferred into three fresh T225 flasks (Corning, New York, NY, USA) containing a combined volume of 150 mL of the same medium, which was additionally EV‐depleted by tangential flow filtration (TFF) with a KrosFlo KR2i RPM System (Repligen, Boston, MA, USA) through a 500 kDa MWCO membrane (Repligen). Cells were treated for 48 h with Creno and/or HPI‐1 in the following concentrations: 0.10% DMSO control, 100 nM Creno + 0.10% DMSO, 2000 nM HPI‐1 + 0.10% DMSO, and 10 nM Creno + 50 nM HPI‐1 + 0.10% DMSO, respectively. Additionally, MOLM‐14 cells were cultured in 15 mL of the same TFF‐treated medium supplemented with either 10 nM Creno + 0.10% DMSO or 50 nM HPI‐1 + 0.10% DMSO. Cells treated with 0.10% DMSO served as control in all cases.

For further proteome analysis, cells were washed three times thoroughly with PBS before being frozen in liquid nitrogen. The conditioned media (CM) of these cells were obtained by centrifugation at 300 × *g* for 5 min and 3000 × *g* for 10 min to remove cells and cell debris, respectively, and cryopreserved at −80°C. EVs were purified from 150 mL of CM as follows: CM was concentrated about 100‐fold by TFF employing a 300 kDa MWCO membrane (both Repligen) to a volume of approx. 1.5 mL. After each purification step the system was washed thoroughly with a basic washing solution (0.50 M NaOH, 154 mM NaCl) and was subsequently re‐equilibrated with neutral buffer (10 mM HEPES pH 7.4, 154 mM NaCl). Subsequently, the concentrated EV samples were further purified by means of size‐exclusion chromatography (SEC), using a qEV2 column (70 nm, Izon Science Limited, Lyon, France) as previously described (Wolf et al. 2022; Nguyen et al. 2020). After the void volume had passed, fractions of 2 mL were collected and individually characterized. EV size and concentration was assessed by tunable resistance pulse sensing (TRPS; Izon Science Limited), whereas protein content was determined by a Bradford assay (Sigma‐Aldrich). Based on this data, EV containing fractions two and three were pooled for further investigations. All cell culture experiments that were used as basis for further cellular and vesicular proteome analysis were conducted as three biological replicates. For HPLC‐MS EV samples were further concentrated by ultracentrifugation. Detailed characterization of EV preparations was performed following the MISEV guidelines (Welsh et al. 2024) and results are summarized in a standardized format according to EV checklist (Poupardin et al. 2024) (Supporting Information: File S1). Human patient EVs were purified from plasma obtained from venous blood of five high‐risk AML patients and four healthy donors from the same cohort as described in (Binder et al. 2021). Briefly, EVs were isolated from 5 mL plasma using 70 nm SEC columns (qEV10; Izon Science) in 3 mL fractions. Particle content was determined using TRPS and the three fractions with highest particle content (fraction range #2–#5) were pooled and used for further experiments.

### 2.4 Proteome Analysis

EV fractions obtained from AML cell lines corresponding to 25 µg of protein were concentrated by ultracentrifugation (100,000 × *g* for 2.5 h). The supernatant was removed, and the remaining pellet was processed by micro S‐traps (Protifi, Huntington, NY, USA) as previously described (Wolf et al. 2022). AML cell line cell pellets as well as CM itself were prepared by mini S‐trap columns (Protifi) relying on the same workflow. EV fractions obtained from high‐risk AML patients and healthy donors were prepared by the same protocol applying micro S‐trap columns (Protifi) as previously described (Wolf et al. 2022). In all cases, obtained tryptic peptides were labelled by tandem mass tags (TMT) as described below.

### 2.5 TMT‐labelling

For cellular proteomics of cell line samples, two sets of TMT 10‐plex were created using a TMT 10plex kit (Thermo Fisher Scientific) according to the manufacturer's instructions. The available TMT‐channels were selected as follows: Channel 126 served as bridge channel containing equal amounts of all 18 samples. TMT‐set 1: 0.10% DMSO control (127N (replicate 1), 127C (replicate 2), and 128N (replicate 3)); 100 nM Creno + 0.10% DMSO (128C (replicate 1), 129N (replicate 2), 129C (replicate 3)); 2000 nM HPI‐1 + 0.10% DMSO (130N (replicate 1), 130C (replicate 2), 131N (replicate 3)). TMT‐set 2: 10 nM Creno + 2000 nM HPI‐1 + 0.10% DMSO (127N (replicate 1), 127C (replicate 2), 128N (replicate 3)); 10 nM Creno + 0.10% DMSO (128C (replicate 1), 129N (replicate 2), 129C (replicate 3)); and 50 nM HPI‐1 + 0.10% DMSO (130N (replicate 1), 130C (replicate 2), 131N (replicate 3)). For visualization of the EV enrichment workflow, cellular, EV, and CM proteomics samples (0.10% DMSO control) were labeled by a TMT 16‐plex kit (Thermo Fisher Scientific) and combined in a second TMT experiment: EVs (126 (replicate 1), 127N (replicate 2), and 127C (replicate 3)); cellular proteome (132C (replicate 1), 133N (replicate 2), and 133C (replicate 3)); conditioned medium (134N (replicate 1), 131N (replicate 2), and 131C (replicate 3)). Samples for comparative cell line EV proteomics were combined in a third TMT experiment using a TMT 16‐plex kit as follows: 0.10% DMSO control (126 (replicate 1), 127N (replicate 2), and 127C (replicate 3)); 100 nM Creno + 0.10% DMSO (128N (replicate 1), 128C (replicate 2), 129N (replicate 3)); 2000 nM HPI‐1 + 0.10% DMSO (129C (replicate 1), 130N (replicate 2), 130C (replicate 3)); and 10 nM Creno +50 nM HPI‐1 + 0.10% DMSO (131N (replicate 1), 131C (replicate 2), 132N (replicate 3)).

For primary EV proteomics of AML patients and healthy donors, peptide samples were labelled by a TMT 16‐plex kit (Thermo Fisher Scientific). As the sample amount in the primary EVs was limited, six of the above mentioned cell line‐derived EVs were additionally pooled in the experiment to boost the number of quantifiable proteins (Woo et al. 2021; Karmani et al. 2024). The labelling was as follows: pool of all included samples (126), cell line‐derived EVs of 0.10% DMSO treatment (127N (replicate 1), 127C (replicate 2) and 128N (replicate 3)); cell line‐derived EVs of 10 nM Creno +50 nM HPI‐1 + 0.10% DMSO treatment (128C (replicate 1), 129N (replicate 2) and 129C (replicate 3)); high‐risk AML patient‐derived EVs (130N (donor 1), 130C (donor 2), 131N (donor 3), 131C (donor 4), 132N (donor 5)); healthy donor‐derived EVs (132C (donor 1), 133N (donor 2), 133C (donor 3), 134N (donor 4)).

### 2.6 HPLC and MS Analysis

The two multiplexed sets of cellular proteome and the multiplexed EV proteome of AML cell line samples, respectively, were subjected to concatenated high‐pH reversed‐phase fractionation (Delmotte et al. 2007). Separation at high pH was carried out on a Gemini NX‐C18 column (150 × 2.0 mm i.d., 3 µm particle diameter, 110 Å pore size; Phenomenex Inc., Aschaffenburg, Germany). Eluent A consisted of H_2_O + 20 mM ammonium formate at pH 10.0 and eluent B of 90% acetonitrile (ACN) + 20 mM ammonium formate also at a pH of 10.0. The multiplexed EV peptides were separated by a multi‐step linear gradient of 1.0% B for 15.0 min, 1.0%–25% B in 170 min, 25%–60% in 60 min, 80% B for 30 min and 1.0% B for 40 min. A total of 100 µL of sample corresponding to 60 µg of peptides were injected. For the two multiplexed cellular proteomes the gradient was altered as follows: 1.0% B for 15 in, 1.0%–30% B for 170 min, 30%–50% for 60 min, 80% B for 30 min and 1.0% B for 40 min. One‐hundred micrograms of peptides were injected in a volume of 100 µL. An automatic fraction collector was used to obtain 24 and 30 fractions for the EV and cellular proteomes, respectively. Samples were pooled into five samples (EVs) and six samples (cells) employing sample concatenation as described by (Yang et al. 2012). Pooled samples were dried in a vacuum centrifuge and resuspended in H_2_O + 0.10% FA to a concentration of approximal 2.0 mg/mL.

Then, samples were measured by low‐pH reversed‐phase‐HPLC employing a µPAC C18 separation column (2000 mm bed length) (PharmaFluidics, Ghent, Belgium) on a nanoHPLC instrument (UltiMate U3000 RSLCnano; Thermo Fisher Scientific). In all cases, the fractionated samples as well as the non‐fractionated samples were measured once. The cellular proteome‐dataset was acquired on a trap system comprising a matching µPAC trap column µPAC C18 trapping column (10 mm bed length.) (Pharmafluidics). The sample was loaded onto the trap by the dedicated loading pump at a flow rate of 4.0 µL/min under isocratic conditions (1.0% ACN, 0.10% TFA) at 50°C. After 10 min, a switching valve was used to connect the trap and analytical column to the nano pump operated at 300 nL/min. A multi‐step gradient employing solvents A (H_2_O + 0.10% FA) and B (ACN + 0.10% FA) was programmed as follows: 1.0% B for 15 min, 1.0%–3.0% B in 15 min, 3.0%–21% in 250 min, 21%–40% in 65 min, 80% B for 15 min and 1.0% B for 60 min. The switching valve was put into its initial position at 410 min.

The comparative EV‐enrichment proteomics and the EV‐proteomics datasets were measured without the trap column at a flow rate of 300 nL/min to increase coverage of polar and small peptides. In addition, the gradient length was increased to obtain maximum protein coverage: 1.0%–21% B in 500 min, 21%–40% in 120 min, 80% B for 20 min and 1.0% B for 60 min.

The primary EVs derived from high‐risk AML patients and healthy donors were analyzed by low‐pH reversed‐phase‐HPLC employing an Aurora Ultimate XT C18 UHPLC column (250 mm × 0.075 mm i.d.; ionopticks, Collingwood, Australia) on a UHPLC instrument (Vanquish Neo, Thermo Fisher Scientific). The separation of 3 µL sample was carried out at 50°C at a flow rate of 300 nL/min. A multi‐step gradient employing solvents A (H_2_O + 0.10% FA) and B (ACN + 0.10% FA) was programmed as follows: 1.0% B for 10 min, 1.0%–10.0% B in 5.0 min, 10.0%–35.0% B in 355 min, 80.0% B for 10 min and 1.0% B for 20 min.

For the AML cell line samples, the nanoHPLC system was hyphenated to a Q Exactive Plus Hybrid quadrupole‐Orbitrap mass spectrometer via a Nanospray Flex ion source (both from Thermo Fisher Scientific). The source was equipped with a SilicaTip emitter with 360 µm o.d., 20 µm i.d. and a tip i.d. of 10 µm (New Objective, Woburn, MA, USA). The spray voltage was set to 1.5 kV, S‐lens RF level to 55 and capillary temperature to 320°C. MS^1^ scans were recorded for *m/z* 350–2000 with a resolution of 70,000 at *m/z* 200. The automatic gain control (AGC) target was set to 3e6 with a maximum injection time of 120 ms. Fifteen data‐dependent MS^2^ scans were obtained for *m/z* 200–2000 at a resolution of 35,000 at *m/z* 200. TMT 10‐plex‐ and TMT 16‐plex‐labelled peptides were then fragmented by higher‐energy collisional dissociation (HCD) at a normalized collision energy of 32 and 30, respectively in an isolation window of *m/z* 1.2. The AGC target was set to 2e5 with a maximum injection time of 250 ms. *m/z* 110.0 was chosen as a fixed first mass for MS^2^ scans. Already fragmented precursor ions were excluded for 30 seconds from further measurement. The instrument was calibrated with Pierce LTQ Velos ESI Positive Ion Calibration Solution (Life Technologies, Vienna, Austria). The sample was measured in three technical replicates. Data acquisition was conducted using Thermo Fisher Scientific Chromeleon 7.2 CDS.

For the primary EVs derived from high‐risk AML patients and healthy donors the nanoHPLC instrument was hyphenated to a QExactive Hybrid quadrupole‐Orbitrap mass spectrometer (Thermo Fisher Scientific). The spray voltage was set to 1.6 kV, S‐lens RF level to 60.0 and capillary temperature to 250°C. The sample was analyzed two times using the same settings for MS^2^, but different *m/z* windows for MS^1^ scans: In one run, MS^1^ scans were recorded for *m/z* 350–2000, in the second run from *m/z* 500–2000, both at a resolution of 70,000 at *m/z* 200. The automatic gain control (AGC) target was set to 3e6 with a maximum injection time of 120 ms. Each MS^1^ scan was followed by 15 data‐dependent MS^2^ scans obtained at a resolution of 35,000 at *m/z* 200. The selected precursor ions were fragmented by higher‐energy collisional dissociation (HCD) at a normalized collision energy of 32 and 30, respectively in an isolation window of *m/z* 1.2. The AGC target was set to 2e5 with a maximum injection time of 250 ms. *m/z* 110.0 was chosen as a fixed first mass for MS^2^ scans. Already fragmented precursor ions were excluded for 30 s from further measurement. The instrument was calibrated with Pierce LTQ Velos ESI Positive Ion Calibration Solution (Life Technologies, Vienna, Austria). Data acquisition was conducted using Thermo Fisher Scientific Chromeleon 7.2.10 ES.

### 2.7 Data Evaluation and Bioinformatic Analysis

Proteomics data were evaluated using MaxQuant software (2.6.3.0; (Cox and Mann 2008)) in default settings correcting for isotope impurities in TMT and TMTpro reagents, as provided by the manufacturer. Protein searches were performed against a Uniprot database, including solely Swiss‐Prot entries for *Homo sapiens* (access: 22.07.2024). Identified protein groups were further processed in Perseus (1.6.14.0, (Tyanova et al. 2016)). Protein abundances were log_2_ transformed, then proteins marked as potential contaminants, those only identified by site, and reverse sequence matches were removed. For enrichment analysis, contaminant proteins were not excluded from further analysis. Data was cleaned from proteins that were not quantified by reporter ions throughout all channels and subsequently median corrected. Further data evaluation was performed with R (version: 4.4.0) (R: A Language and Environment for Statistical Computing 2024). Differences in expression were calculated by applying the LIMMA package with default parameters (Ritchie et al. 2015) and *p* values were adjusted for multiple testing with Benjamini–Hochberg correction. Gene identifiers were downloaded from Uniprot for the proteins analyzed. Fast gene set enrichment analysis was performed on proteins ordered by log fold change with the FGSEA package (Korotkevich et al. 2016). The KEGG_2021_Human database was downloaded from Enrichr (Xie et al. 2021). KEGG pathways were visualized using the pathview package (Luo and Brouwer 2013). Pathways associated with diseases and infections were removed from figures displaying pathway analysis results. For statistical analysis, one‐way ANOVA with Dunett's post hoc test, two‐way ANOVA with Greenhouse–Geisser correction and with Dunett's post hoc test and Limma with Benjamini–Hochberg correction were used as appropriate, and the respective statistical analysis is indicated in the figure caption.

For contextualization, previously published data on AML cell line‐EVs were analyzed (table S1 of Kang et al. (2021)), re‐evaluated and set into the perspective of the current study. Precisely, the protein intensities from the EV proteomes of three AML cell lines (KG‐1, HL‐60 and THP‐1) and two non‐AML cell lines (human dermal fibroblasts [HDFa] and human mensenchymal stromal cells [hMSCs]) here, were log2‐transformed, normalized by subtracting the median intensity, and finally subjected to differential analysis using Limma in R as described above.

## 3 Results

### 3.1 Combined Crenolanib and HPI‐1 Treatment Synergistically Inhibit AML Cell Metabolism and Proliferation

**FIGURE 1:**
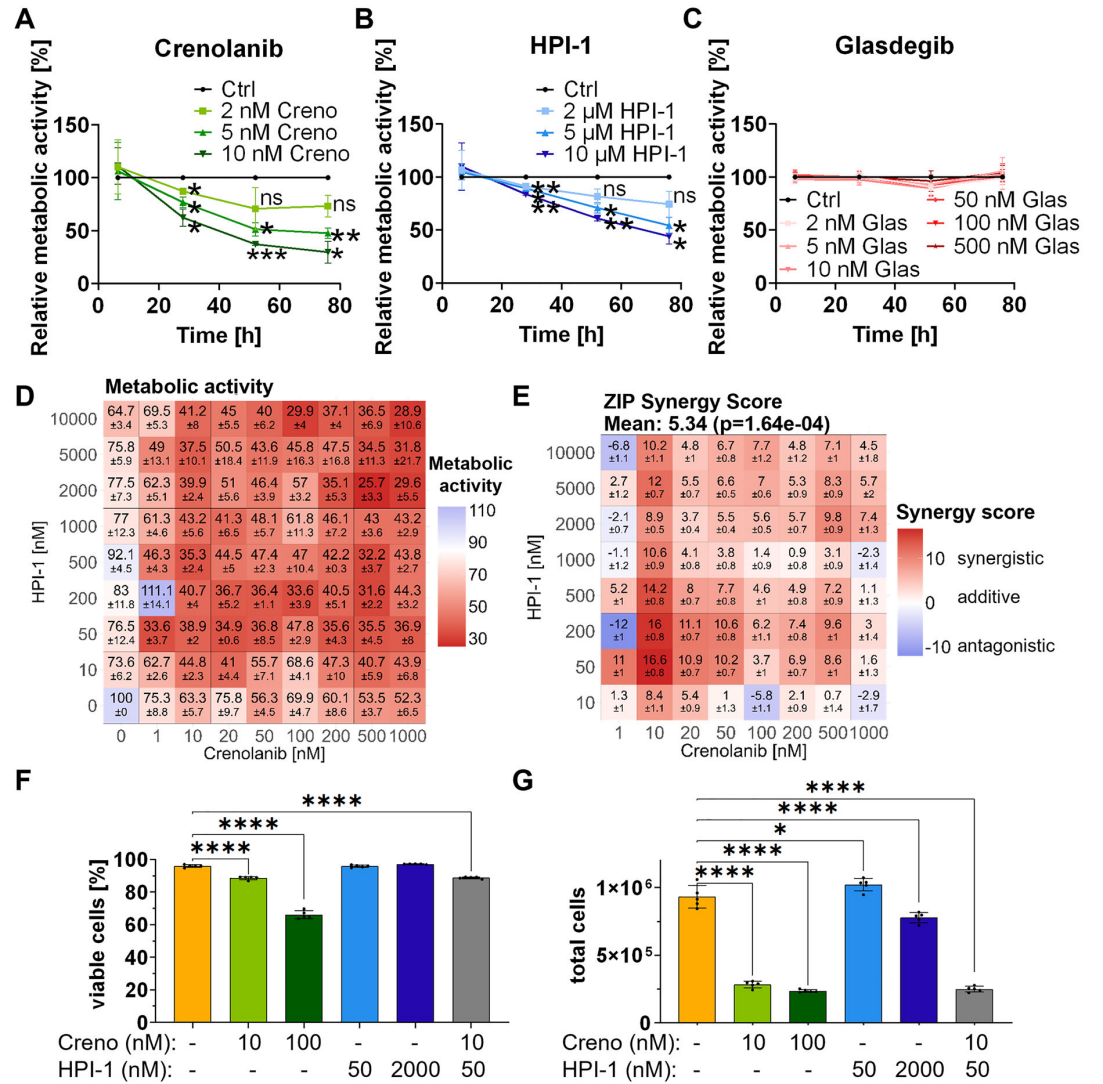
Combined inhibition of FLT3 and Hh signalling reduces cell metabolic activity and proliferation of MOLM‐14 AML cells. The effect of (A) Creno, (B) HPI‐1, (C) Glas on the metabolic activity of MOLM‐14 cells was monitored during a time span of 78 h. Varying concentrations of (A) 2.0, 5.0, and 10 nM Creno, (B) 2.0, 5.0, 10 µM HPI‐1 and (C) 2.0, 5.0, 10, 50, 100 and 500 nM Glas were tested compared to the 0.10% DMSO‐treated control cells. Experiments were performed as biological triplicates, each consisting of three technical replicates. Differences were calculated using two‐way ANOVA with Greenhouse–Geisser correction and Dunett's post‐hoc test. (D) Metabolic activity after 30 h in % compared to the control and normalized to a treatment period of 7 h is depicted. Mean values and standard deviations are given (*n* = 3). (E) An isobole analysis of Creno and HPI‐1 treatment of MOLM‐14 cells was performed based on the data from (D) to determine synergistic and antagonistic effects. Synergistic (red) and antagonistic (blue) effects were calculated according to the zero interaction potency (ZIP) independence model (*n* = 3). Cell viability (F) and proliferation (G) were assessed after 48 h treatment with either Creno, HPI‐1 or a combination of both as indicated. Data are displayed as mean ± standard deviation from five biological replicates. Differences were calculated by one‐way ANOVA with Dunett's post‐hoc test. (*p ≤ 0.05, **p ≤ 0.01, ***p ≤ 0.001 and **** *p* ≤ 0.0001), n.s. for non‐significant.

In order to assess the response of AML cells to targeted pathway inhibition, the two different FLT3‐ITD‐mutated AML cell lines MOLM‐14 and MV4‐11, with reported GLI expression (Lim et al. 2015), were exposed to different concentrations of Creno, HPI‐1 and Glas. Administration of different concentrations of Creno (Figures 1A and S1A) and HPI‐1 (Figures 1B and S1B) curbed the metabolic activity of both cell lines in a dose‐ and time‐dependent manner, as measured by resazurin reduction to resorufin. Glas had no significant effect on the metabolic activity of either cell line (Figures 1C and S1C).

Next, we tested for synergistic or antagonistic effects of Creno and HPI‐1. Figures 1D and S1D show the percentage of metabolic activity after 30 h of treatment for MOLM‐14 and MV4‐11 cells, respectively. This data served as input to calculate synergy relying on the ZIP algorithm (Figures 1E and S1E). Calculated synergy score values correspond to the change in potency between individual drugs and their combinations. In both cell lines, we found that lower concentrations of Creno and HPI‐1, that is, 1–50 nM Creno and 10–1000 µM HPI‐1, acted synergistically on cell metabolic activity. Antagonistic effects were weak and solely observed for very low concentrations of either Creno or HPI‐1. Since proteomics experiments were performed with cells that were grown in medium that was tangential flow‐filtered (TFF) to remove serum‐derived particles including EVs and protein aggregates, we additionally measured synergistic effects in this medium. Previously observed synergistic effects in normal medium (Figure 1D,E) were even more pronounced when cells were cultured in media after EV‐depletion (Figure S2A,B).

Since ZIP synergy values peaked at 10–50 nM Creno and 50–500 nM HPI‐1, we next analyzed AML cell viability and proliferation at the lowest synergistic dose, which notably also produced the highest synergy score: 10 nM Creno with 50 nM HPI‐1 (Figure 1E). In addition, we also used single treatments at equal or higher concentrations of pathway inhibitors (10 nM/100 nM Creno and 50 nM/2000 nM HPI1). Among the single treatments, Creno decreased both cell viability and the total cell number in a dose‐dependent manner. HPI‐1 had no effect on cell viability, but treatment with 2000 nM HPI‐1 decreased cell proliferation. The combined AML cell treatment using 10 nM Creno and 50 nM HPI‐1 slightly reduced cell viability and strongly inhibited cell proliferation (Figure 1F,G). Taken together, these findings indicate that while the Creno–HPI combination synergistically inhibits AML cell metabolism, Creno alone predominantly drives the reduction in cell viability and proliferation, suggesting that its effects in these parameters may predominate over those of the combination.

### 3.2 Proteomics of MOLM‐14 cells after combined pathway inhibition with Creno and HPI‐1 reveals impact on cellular metabolism

**FIGURE 2:**
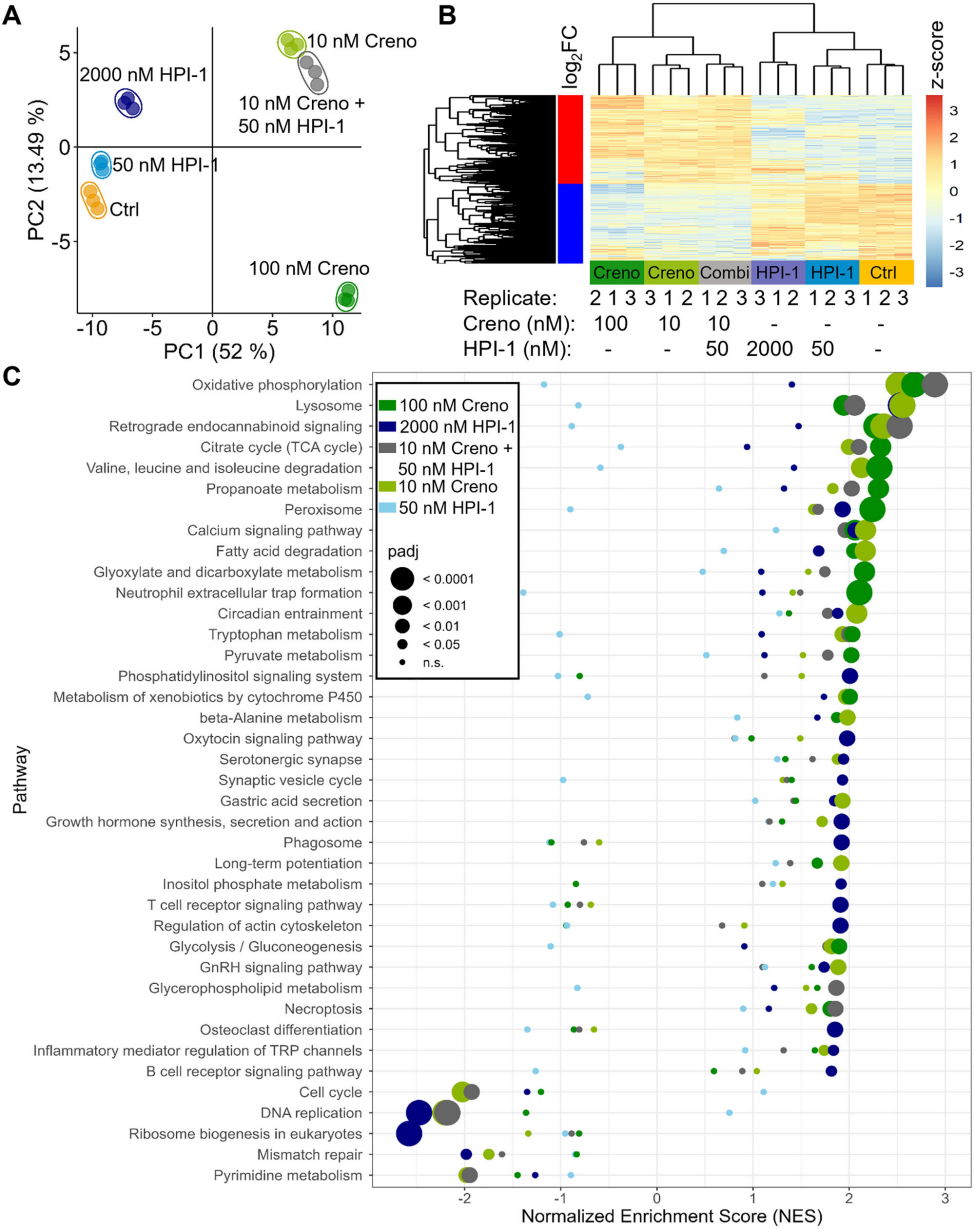
Multiplexed proteomics of the cellular proteome of MOLM‐14 cells after pathway inhibition reveals impact on cellular metabolism in Creno and HPI‐1 treated AML cells. (A) PCA based on the 5520 proteins quantified throughout the six conditions and their biological triplicates. Treatments were coloured as follows: 0.10% DMSO‐treated control (yellow), 50 nM HPI‐1 (light blue), 2000 nM HPI‐1 (dark blue), 10 nM Creno (light green), 100 nM Creno (green) and 10 nM Creno + 50 nM HPI‐1 (grey). (B) Comparison of expression levels (z‐scores of normalized intensities) of significantly up‐ or downregulated proteins by treatment with 10 nM Creno + 50 nM HPI‐1 in comparison to the 0.10% DMSO control treatment with levels of those of other treatments. (C) Pathway analysis of all treatments in comparison to the control. Pathways achieving NES ←1.8 or >1.8 and a *p* value of ≤ 0.05 for any of the five different treatments are shown in the plot.

To gain insights into the molecular mechanisms contributing to the observed synergy effect, we aimed at comparing the cellular proteomes of MOLM‐14 cells treated with the different concentrations of pathway inhibitors. For this purpose, cells were treated with the previously selected concentrations of Creno and/or HPI‐1 for 48 h in EV‐depleted medium. Cell samples from three biological replicates were then collected, TMT‐labelled and multiplexed using a bridge channel. The proteomics workflow is shown in Figure S3A. To identify a maximum number of proteins, samples were fractionated by high‐pH reversed‐phase chromatography (Delmotte et al. 2007) and after fraction concatenation, separated by low‐pH reversed‐phase chromatography coupled to quadrupole‐Orbitrap mass spectrometry operated in DDA mode. Only proteins showing quantitative data in all TMT channels were considered for further analysis, resulting in 5520 identified proteins. Principal component analysis (PCA) revealed strong clustering of the biological replicates and distinct protein expression profiles of the different treatment groups (Figure 2A). The expression intensities of the 3176 significantly up‐ or downregulated proteins in cells treated with a combined treatment compared to the control sample are plotted in Figure 2B. Interestingly, the expression profile of cells treated with Creno and HPI‐1 differed from the profile of 0.10% DMSO control and HPI‐1 only treated cells, but closely resembled Creno treated cells (Figure 2A,B). Of note, throughout all samples we identified FLT3 expression; however, we could not detect GLI1 or GLI2 expression.

To assign the large number of identified proteins and their respective expression values to pathways, we performed fast gene set enrichment analysis (FGSEA) as previously described (Korotkevich et al. 2016). We used the KEGG database as a reference for pathway analysis. Figure 2C shows significantly altered canonical pathways identified by FGSEA, ranked by their normalized enrichment score (NES). Pathways with a negative NES are downregulated, whereas a positive NES represents upregulation. A detailed table with all identified pathways and their corresponding NES and *p* values can be found for all different treatments in the supplement (Table S1).

When filtering with the above criteria, treatment with a combination of Creno and HPI‐1 resulted in 19 altered pathways. Treatment with 50 nM and 2000 nM HPI‐1 resulted in 1 and 27 significantly differentially regulated pathways, respectively, and treatment with 10 and 100 nM Creno altered 26 and 22 pathways, respectively. Treatment with Creno and the combined treatment resulted in a prominent upregulation of oxidative phosphorylation (OXPHOS) and pathways fuelling into mitochondrial respiration, such as TCA cycle activity, degradation of fatty acids and the branched chain amino acids, valine, leucine and isoleucine (Figure 2C). A downregulation of ribosomal biogenesis was observed with HPI‐1 treatment, as well as a reduction in DNA replication, which was also observed with Creno treatment and the combined treatment (Figure 2C). The close proximity of the cellular proteomes of MOLM‐14 cells treated with only 10 nM Creno and treated with 10 nM Creno in combination with 50 nM HPI‐1 in the PCA (Figure 2A) suggests the global effect of HPI‐1 on protein expression may be minor, warranting closer examination to clarify its specific contribution.

Therefore, we directly compared protein expression profiles between these two groups (Figure S4). Notably, several of the most downregulated proteins in the combination group were linked to lipid metabolism (APOC3, JARID2, ARSD, PSAP and FADS2). Conversely, and consistent with altered expression of proteins associated with oxidative phosphorylation activity (Figure 2C), multiple mitochondrial‐associated proteins, for example, NDUFB1, CISD1 and SQOR, showed an upregulation. Together, these findings point to a dysregulated metabolic and energy pathway specifically in cells treated with Creno and HPI in combination.

### 3.3 Comparing Proteomes Obtained From Cells, EVs, and CM Highlights Their Differential Protein Content

To determine if the synergistic treatment effect of Creno and HPI‐1 on AML cells can influence their surroundings via EVs, we analyzed the EV proteome to identify proteins or protein groups selectively transported or excluded from vesicles after treatment. We first compared the proteome of control cells with the proteome of their EVs and the respective CM. EVs isolated from treatments yielded approximately the same amount of EVs after purification with TFF and SEC and were in the range of 1.8–5.2·10^9^ particles per mL (Figure S3B). In addition, they showed no difference in size.

**FIGURE 3:**
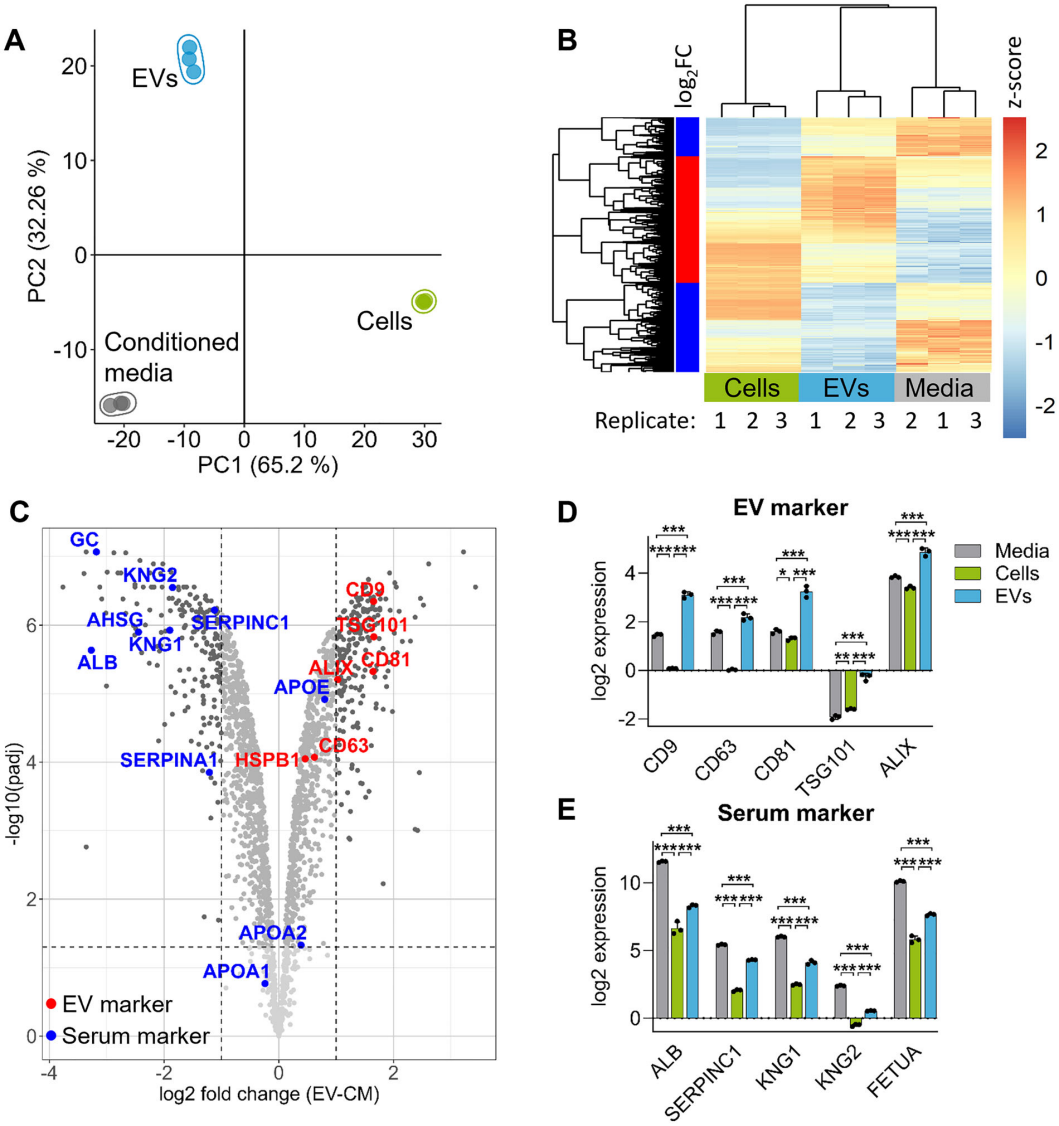
Comparison of the proteomes obtained from cells, EVs and CM. (A) PCA of the proteomes of cells, EVs and CM from 0.10 % DMSO‐treated control cells. (B) Comparison of expression levels (z‐scores of normalized intensities) of significantly up‐ or downregulated proteins between EVs and CM contrasted to respective expression levels of the cellular proteome. (C) Volcano plot depicting significantly altered proteins between the EV proteome and CM. Dots represent mean of three biological replicates. EV markers (red) and serum proteins (blue) are marked. (D and E) Bar charts comparing relative abundances of selected EV marker proteins (D) and serum marker proteins (E). Bars represent mean ± standard deviation of three biological replicates, differences were calculated with LIMMA and results were corrected for multiple testing with the Benjamini–Hochberg procedure. *p < 0.05; **p < 0.01; ***p < 0.001.

First, we compared the proteome of DMSO‐treated control cells to the vesicular proteome and the proteome of the CM. The PCA demonstrated that the three proteomes clearly differ from each other (Figure 3A). Analysis of differential regulation between conditioned media and EVs revealed 1699 up‐ or downregulated proteins, which corresponds to more than 80% of all identified proteins. The expression profiles of these significantly regulated proteins differed, often greatly, also from the cellular proteome (Figure 3B). Investigation of differential regulation between proteins revealed that well‐known EV markers such as the tetraspanins CD9, CD63, CD81, the programmed cell death 6‐interacting protein (PDCD6IP, ALIX) and tumor susceptibility gene 101 (TSG101) which are involved in sorting cargo to EVs are enriched in the EV samples whereas bovine serum proteins such as albumin, vitamin D binding protein (GC) or kininogen1 and 2 are found in relatively higher values in the CM (Figure 3C). Also, the comparison of the expression levels of EV markers and serum markers confirmed an enrichment in the respective samples (Figure 3D,E). Importantly, as CM represents the supernatant of the cultivated cells, these samples contain also EVs and therefore EV markers. Conversely, serum proteins can adhere to cells and EVs by forming a protein corona (Wolf et al. 2022; Toth et al. 2021), appearing at lower levels in non‐CM samples.

### 3.4 Distinct vesicular protein content of MOLM‐14 cells after pathway inhibition

**FIGURE 4:**
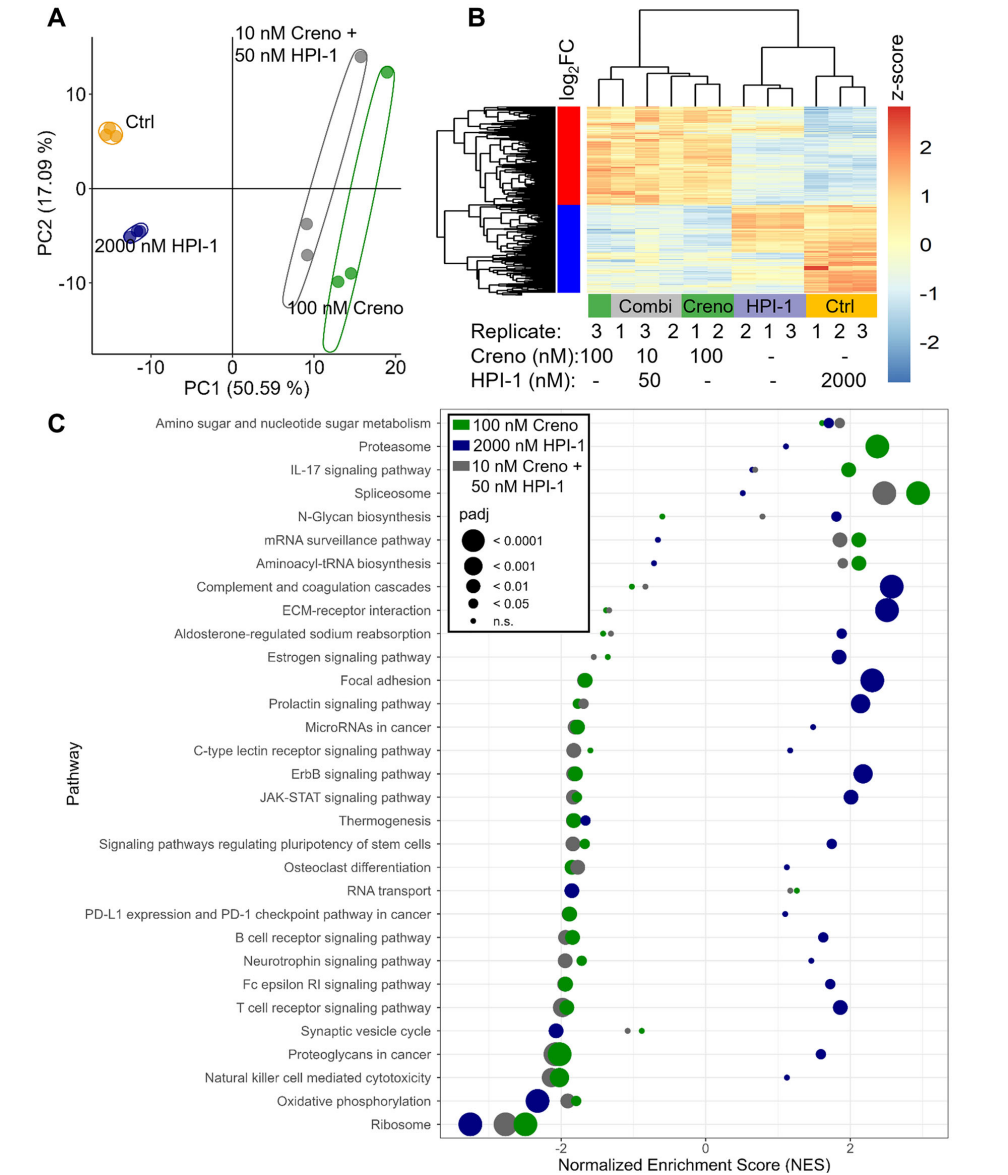
Proteomics of the vesicular proteome of MOLM‐14 cells after pathway inhibition. (A) PCA based on the 3475 proteins quantified throughout the four conditions and their biological triplicates. Treatments were coloured as follows: 0.10% DMSO‐treated control cells (yellow), 2000 nM HPI‐1 (blue), 100 nM Creno (green) and 10 nM Creno + 50 nM HPI‐1 (grey). (B) Comparison of expression levels (z‐scores) of significantly up‐ or downregulated proteins upon treatment with 10 nM Creno + 50 nM HPI‐1 in comparison to 0.10% DMSO control with levels of those of the other treatments. (C) Pathway analysis of all treatments in comparison to the control. Pathways that have a NES < ‐1.8 or > 1.8 and a *p* value of < 0.05 for any of the four different treatments are shown in the plot.

Next, we investigated the influence of treatment with Creno, HPI‐1 or the combinational treatment on the vesicular proteome. Based on the synergy data from our initial analysis, we decided to investigate the following four conditions: the 0.10% DMSO control, incubation with either 100 nM Creno or 2000 nM HPI‐1 and the combination of both drugs at concentrations of 10 nM Creno and 50 nM HPI‐1. We identified 3476 proteins across all samples. PCA revealed clustering of the biological replicates of the different treatments (Figure 4A). Treatment with the combination of both Creno and HPI‐1 resulted in the significant differential expression of 1577 proteins in the EV proteome in comparison to the control (Figure 4B). The PCA and the expression profile revealed a strong correlation of the combinational treatment with single treatment with the Creno single treatment, however, it is greatly different from HPI‐1 single treatment (Figure 4A,B).

Again, we assigned the regulated proteins into pathways using FGSEA and the KEGG pathway library and used stringent filtering: NES > 1.8 or < ‐1.8, *p* value < 0.05 (Figure 4C). When filtering with these criteria, treatment with a combination of Creno and HPI‐1 altered 31 pathways, treatment with 2000 nM HPI‐1 resulted in 26 significantly differentially regulated pathways, and treatment with 100 nM Creno caused 23 altered pathways, respectively. Protein members of different pro‐proliferative signalling pathways such as ErbB signalling pathway and JAK‐STAT signalling pathway were downregulated by Creno and combined treatment whereas similar pathways were upregulated by HPI‐1 treatment (Figure 4C). Additionally, Creno and the combined treatment caused upregulation of the spliceosome and proteasome in comparison to the 0.10 % DMSO control (Figure 4C). OXPHOS proteins, upregulated by Creno and combinational treatment in the cellular proteome (Figure 2C), was downregulated in the vesicular proteome of all three treatments in comparison to the control (Figure 4C).

### 3.5 Comparison of the cellular and the vesicular proteome after pathway inhibition

**FIGURE 5:**
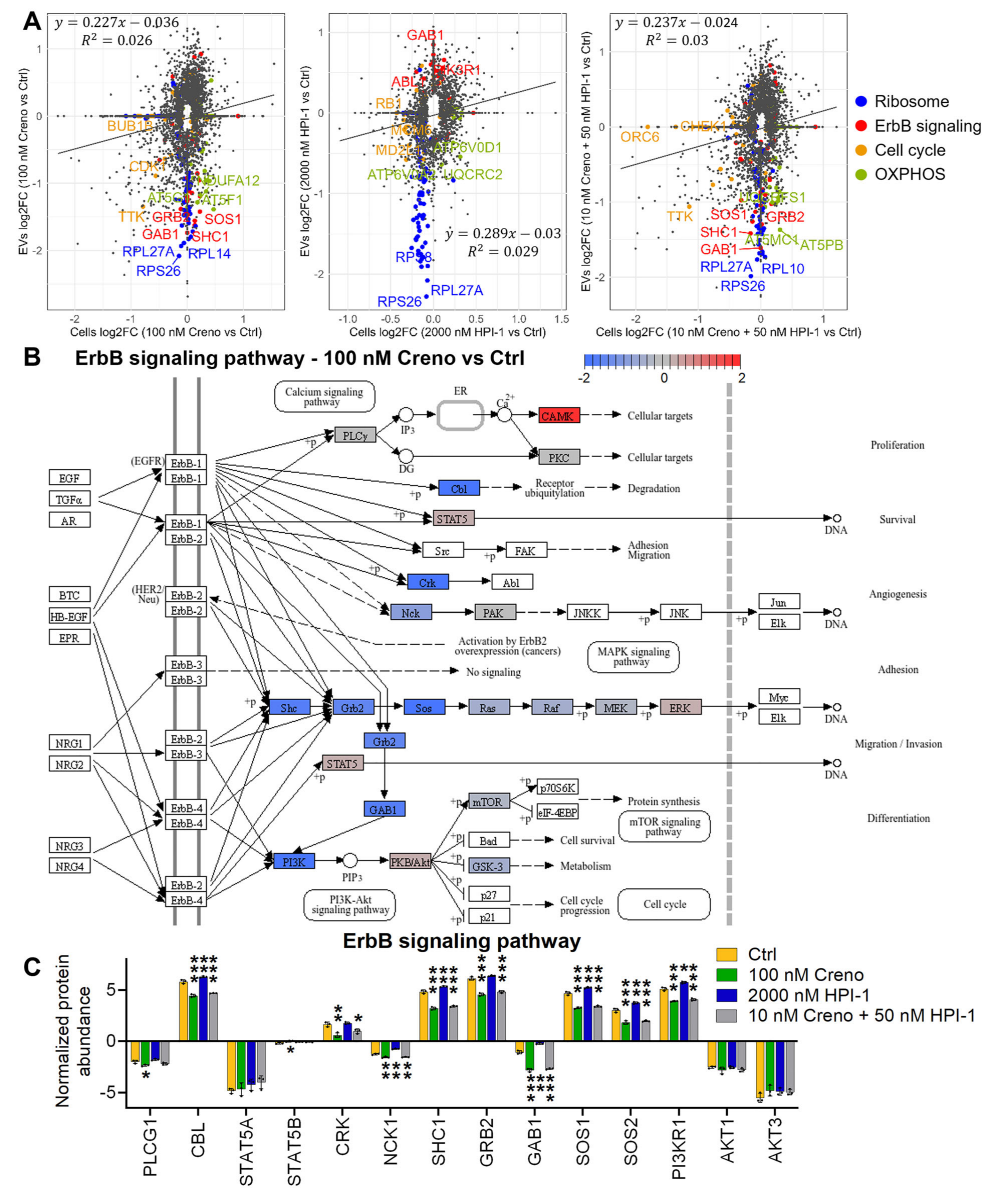
Proteins involved in ErbB signalling are downregulated in the EV but not in the cellular proteome after Creno treatment. (A) Correlation of protein abundance changes at EV and cellular proteome levels upon indicated treatments. Proteins significantly regulated in either of the proteomes are displayed. Proteins assigned to previously identified, top‐scoring pathways were marked as follows: ribosome (blue), ErbB signalling (red), cell cycle (orange), OXPHOS (green). (B) ErbB signalling as described by KEGG (hs04012) was coloured based on expression levels comparing EVs treated with 100 nM Creno to 0.10% DMSO‐treated control (mean of *n* = 3). Upregulation is shown in red, downregulation in blue. (C) Vesicular expression levels of initial proteins of the ErbB signalling sub‐axes. Bars represent mean ± standard deviation, *n* = 3. Group differences were calculated by LIMMA and corrected for multiple testing by Benjamini–Hochberg method. *p < 0.05; **p < 0.01; ***p < 0.001.

To determine whether treatment with Creno and/or HPI‐1 altered the vesicular and cellular proteomes to a similar extent and in the same direction, we plotted proteins that were significantly different in either the vesicular or cellular proteome compared to the control group against each other (Figure 5A). We were interested in identifying pathways that were highly regulated either in both proteomes or selectively in one proteome but not the other. For all treatments, the cellular and EV proteomes correlated positively. Notably, regardless of the treatment, we found an accumulation of proteins that were downregulated in EVs upon pathway inhibition compared to the control, but had little regulation in the cellular proteome. When we manually annotated the most regulated proteins, we found that many of them could be assigned to the ribosome in all different treatment groups. While the presence of ribosomal proteins in the cellular proteome was unchanged by treatment with different pathway inhibitors, their export to the vesicles was highly decreased (Figures 5A and S5A–C).

In addition, we observed that many key members of the ErbB signalling pathway were highly downregulated in the vesicular proteome after Creno or combined creno and HPI‐1 treatment compared to the untreated control, a pathway that is also unchanged in the cellular proteome but upregulated in the vesicular proteome by HPI‐1 treatment (Figure 5A). ErbB signalling is an important cellular pathway, regulating cell proliferation, survival and migration, among other functions. Downstream of ligand binding to ErbB receptor tyrosine kinases, several different signalling pathways can be activated. The most prominent ones are calcium signalling, MAPK signalling and PI3K‐Akt signalling (Wang 2017). We analyzed the protein expression levels of components of ErbB signalling and identified that especially downstream MAPK‐signalling and PI3K‐Akt signalling were downregulated by Creno treatment (Figure 5B). Similarly to Creno treatment also the combinational treatment downregulated the same axes, however treatment with HPI‐1 alone led to an upregulation of all downstream pathways of ErbB signalling (Figure S6A,B). We additionally picked the starting points of all sub axes of ErbB signalling and plotted their expression levels, showing that Creno and the combinational treatment with Creno and HPI‐1 downregulated most of the pathway members (Figure 5C). As also seen in Figure 5A, components were mostly unchanged in the cellular proteome (Figure S7).

Of note, we discovered 605 proteins uniquely in the vesicular proteome but not in the cellular proteome. Of those 28 are sorted to the KEGG pathway of extracellular matrix‐(ECM) receptor interaction and 31 are related to focal adhesion (Table S2).

To put these findings into the perspective of non‐AML cells, we further evaluated and integrated data from a previous paper by Kang et al. comparing the EV proteomes of AML cell lines to non‐AML cell lines (Kang et al. 2021). As evident from the volcano plot (Figure S8), AML cell lines secrete EVs with highly upregulated ribosomal proteins compared to non‐AML cell lines. In addition, GRB2, an important member of the ErbB signalling family, and PRKDC, involved in cell cycle regulation, were significantly upregulated in AML cell line EVs. Unfortunately, likely due to a lower protein coverage and differences in the EV purification strategy, the general coverage of proteins involved in ErbB signalling, oxidative phosphorylation and cell cycle was limited.

### 3.6 Comparative EV Proteomics in High‐risk AML Patients and Healthy Donors

**FIGURE 6:**
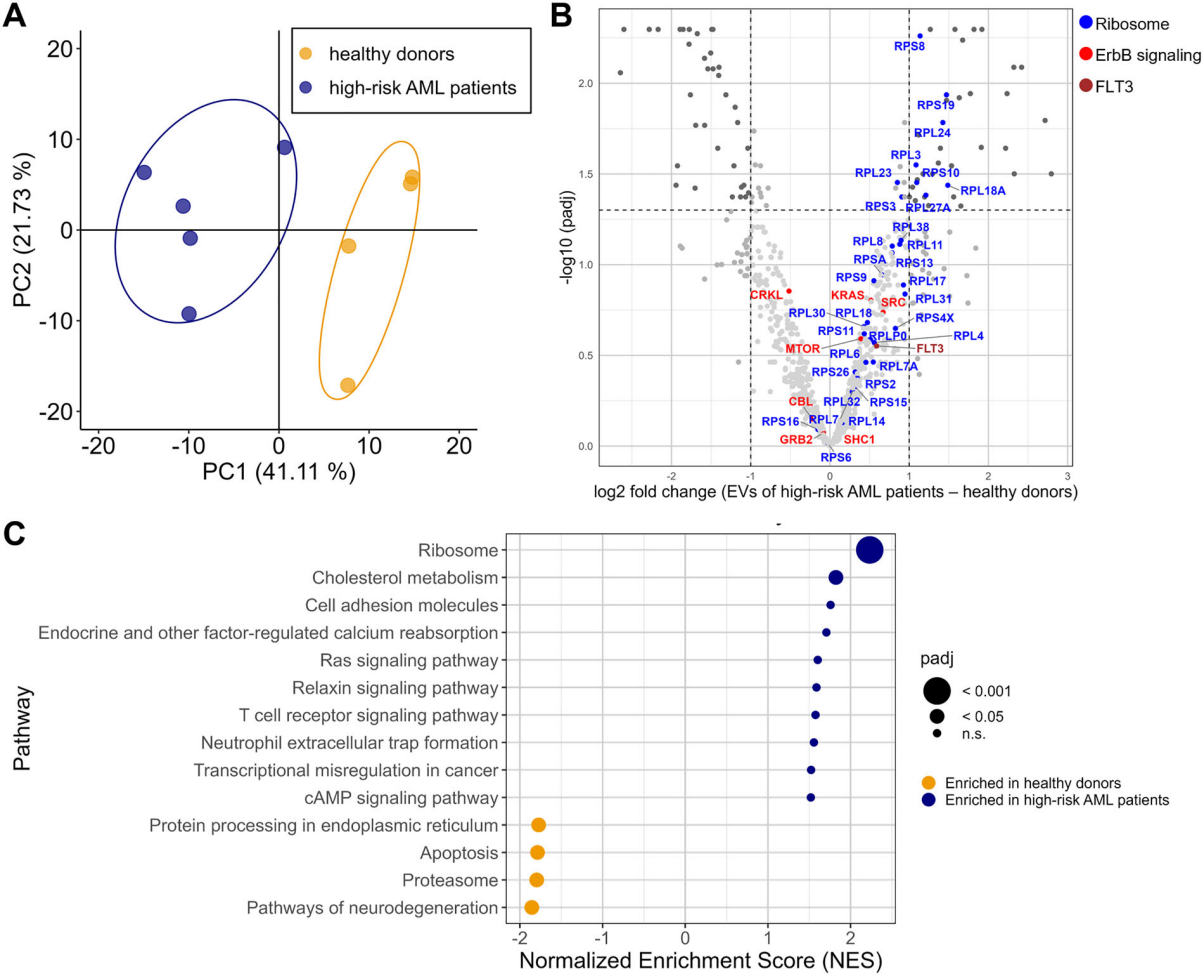
AML patients’ blood EVs display strong alterations in the abundance of ribosomal, FLT3 and ErbB signalling proteins in multiplexed quantitative proteomics. (A) Principal component analysis (PCA) based on the 776 EV‐associated proteins quantified in five high‐risk AML patients and four healthy donors. (B) Volcano plot depicting proteins altered in the EV proteome of high‐risk AML patients compared to healthy donors. Proteins related to the ribosome are coloured in blue, ErbB signalling in red, and FLT3 signalling in brown. (C) Pathway analysis of the proteins altered in high‐risk AML patients compared to healthy controls. Pathways that achieved normalized enrichment scores (NES) < ‐1.5 or > 1.5 are shown in the plot.

To validate if our findings in AML cell lines translate to AML patients, we investigated the proteomes of EVs purified from plasma of high‐risk AML patients and healthy donors as a control. As evident from the PCA plot in Figure 6A, the five high‐risk AML patients could be clearly separated from the four healthy controls based on their differences in the EV proteomes. The differences driving this separation could be largely attributed to a number of ribosomal proteins that were upregulated in the EVs of high‐risk AML patients (Figure 6B). Moreover, all identified proteins assigned to either ErbB‐signalling or FLT3 were enriched in high‐risk AML patients’ EV samples. Notably, the coverage of ErbB‐signalling proteins was limited in comparison to the AML cell line model. Next, alterations in the EV proteomes were grouped into significantly regulated pathways, revealing that several pathways were up‐ or down‐regulated in high‐risk AML patients’ EVs compared to healthy donors (Figure 6C).

## 4 Discussion

The present study revealed synergistic effects of single or combined inhibition of FLT3 with Creno and/or the Hh pathway with Glas and HPI‐1 in the AML cell lines MOLM‐14 and MV4‐11. We discovered a time and dose dependent reduction of cellular metabolic activity upon treatment with Creno and HPI‐1 of both cell lines. In contrast, Glas treatment had no effect on metabolic activity, likely due to its inhibition of SMO at an early step in the Hh pathway, which may be bypassed by non‐canonical Hh signalling (Munchhof et al. 2012). HPI‐1 has been described to target Hh signalling directly via inhibiting the activity of GLI1 and 2 through disruption of their interaction with bromodomain and extra‐terminal (BET) domain protein members (Bagka et al. 2023). However, its broad targeting of BET proteins suggests that the reduced metabolic activity observed with HPI‐1 treatment may not be solely due to GLI inhibition. The reduction in metabolic activity seen upon Creno and HPI‐1 can be explained by a reduction in cell proliferation after HPI‐1 treatment, whereas Creno treatment additionally leads to a reduction in cell viability. This was also observed by cellular pathway analysis, as DNA replication was downregulated by Creno and HPI‐1 treatment. Relatively low concentrations of Creno and HPI‐1 resulted in synergistic effects when combined as treatment in both cell lines. These observed synergistic effects were even more pronounced when the treatment media was previously depleted of EVs, suggesting that media‐ or serum‐derived EVs or proteins might support chemoresistance in AML cells.

As a central aspect of the study, we implemented a comprehensive proteomics approach to provide a multifaceted perspective on how AML cells selectively sort proteins to EVs upon treatment with Creno and/or HPI‐1. Measuring the proteome of EVs is challenging due to the low abundance of EVs in the culture medium and the overall low concentration of vesicular proteins. To detect a maximum number of proteins, we employed off‐line high‐pH reversed‐phase fractionation followed by conventional low‐pH reversed‐phase separation coupled to MS; a strategy that has been reported to increase protein identification 1.6‐fold (Delmotte et al. 2007; Yang et al. 2012). In addition, we utilized extended gradient lengths and a dedicated HPLC column to further maximize the protein coverage of the cellular and vesicular proteomes, respectively. As the proteomics experiments were employing isobaric TMT‐labelling and data‐dependent acquisition, we could combine different treatment groups in one run and boost the signal intensities even further. Following this protocol, we were able to identify 5570 proteins across six conditions in the cellular samples and 3476 proteins in the vesicular samples across four conditions. Compared to the literature this is a respectable result; in a recent study by Cross et al., where they aimed to identify a maximum number of proteins in EVs from SW480 cells, they were able to identify 3988 proteins, however, they used a lower sample amount but only aimed to achieve detection throughout two replicates (Cross et al. 2024).

By achieving a high number of identified proteins in the vesicular proteome, we were also able to uniquely identify 605 proteins, representing 17% of all vesicular proteins detected in the EV proteome. Although these proteins are ultimately also of cellular origin as they need to be produced by the cells, they were only detectable in the vesicular proteome and thus we were able to show active sorting of proteins to the EVs. As EV cargo has long been thought to reflect the protein content of the parent cell, and as this view has only recently changed (Anand et al. 2019; Greening et al. 2024), our study provides additional insight into the active sorting of proteins to EVs. Among the proteins identified only in vesicles, we also identified a large cohort of proteins associated with ECM‐receptor interactions, which is not surprising since interactions with the ECM have been described to be important for EVs, especially for cancer vesicles in the context of survival and metastasis (Greening et al. 2024). We also found an enrichment of proteins associated with focal adhesions in the EV proteome, as previously already reported by Jimenez et al., and vesicular cell–cell adhesion proteins were identified as important for enabling cell adhesion to surfaces (Jimenez et al. 2019).

Although we found that the EV and the cellular proteomes correlate to some extent, proteins related to certain pathways are actively sorted. Proteome analysis revealed that, at the cellular level, treatment with Creno and the combination of Creno and HPI‐1 resulted in a significant upregulation of OXPHOS and pathways that support mitochondrial respiration. However, surprisingly, all treatments led to a reduction of OXPHOS proteins in the vesicular proteome compared to the control. Cells may have a high demand for energy‐producing proteins and be unable to secrete them into recipient cells, or they may send a signal of their current cellular state to neighbouring cells by failing to transport OXPHOS components.

Notably, Creno and the combined Creno and HPI‐1 treatment led to a downregulation of proteins associated with proliferative signalling pathways, such as ErbB and Jak‐Stat signalling. Intriguingly, the amount of these proteins changed significantly in EVs but remained unaltered in the cellular proteome upon treatment. Notably, in ERBB2 overexpressing mammary cells, members of the ErbB pathway were shown to be overrepresented in the EV cargo compared to the cellular content, and this excess was associated with malignant transformation (Amorim et al. 2014). FLT3‐ITD AML cells have been described to be resistant to PI3K/Akt pathway inhibitors due to concomitant STAT5 activation, highlighting the importance of maintaining this pathway (Nogami et al. 2015). Interestingly, ErbB signalling is also mentioned as a potential therapeutic target in leukaemia (Liao et al. 2017). Together with our data, these findings suggest that ErbB signalling pathway members are transported by EVs and their expression is important for oncogenesis. We hypothesize that potentially the reduction of the ErbB signalling pathway members in the EVs following Creno treatment of MOLM‐14 cells could explain in parts the therapeutic success showing that less patients suffer from relapse (Wang et al. 2024). Unfortunately, a cellular or animal model demonstrating that EVs after Creno treatment exhibit antitumorigenic properties, thereby providing direct evidence for our hypothesis, or exhibiting pro‐tumorigenic effects as it is mostly described for chemoEVs (Ab Razak et al. 2019) is not yet available. Crucially, by comparing the EV proteomes of high‐risk AML patients to those of healthy donors, we found that all proteins assigned to ErbB signalling were upregulated in EVs from AML patients, further corroborating our findings. However, insights into ErbB signalling in primary EVs have been somewhat limited due to generally lower coverage compared to the cell line model.

As previously reported, myeloma cells secreted vesicles with enhanced levels of ribosomal proteins compared to healthy controls that were then able to initiate translation in receiving cells and were important for pro‐proliferative effects (Dabbah et al. 2021). Notably, serum starvation, inhibition of export of ribosomal proteins from the nucleus and inhibition of MAPK‐activated p90 ribosomal S6 kinase (RSK) signalling all inhibited production of ribosomal proteins and export to vesicles (Dabbah et al. 2021). This is interesting as we found that the presence of ribosomal proteins in the cellular proteome was unchanged by treatment with different pathway inhibitors and only treatment with HPI‐1 decreased ribosomal biogenesis. However, similar to previous results applying different treatments (Dabbah et al. 2021), addition of Creno, HPI‐1 and a combination of both reduced the export of ribosomal proteins to EVs. This was also in line with our data on EV proteomes obtained from primary patient samples. Healthy donor samples showed significantly lower content of several ribosomal proteins compared to AML patient‐derived EVs. This picture was further supported by the re‐evaluation of the comparison of EV proteomes of AML cell lines and non‐AML cell lines reported by (Kang et al. 2021) (Figure S8): Again, a striking upregulation of ribosomal proteins together with key proteins of the ErbB pathway was found in AML EVs. Thus, EVs from healthy donors rather resemble EVs from AML cell lines treated with FLT3 and/or Hh pathway inhibitors pointing towards a beneficial alteration of EV content upon treatment. This finding could be potentially relevant for diagnostic or predictive purposes during treatment of the disease.

Since the effects observed at the level of cellular proteomics for lower treatment concentrations were less pronounced or comparable to those for the corresponding higher concentrations of Creno and HPI‐1, we omitted these samples from the analysis of the vesicular proteome. However, as the combined treatment revealed very similar effects in terms of up/downregulated pathways compared to the high Creno dose (100 nM), additional measurement of the lower dose of 10 nM Creno would be interesting. In addition, it should be mentioned that although the EVs investigated in this study shared a homology in size, there is a possibility of having two or more subpopulations. As we discovered a certain amount of chemotherapy‐related cell death and a reduction in cell proliferation, there might be distinct EV populations released from cells of differential fate. These populations may include EVs from chemoresistant cells or vesicles from cells undergoing pre‐apoptotic processes and may exhibit different expressions of ErbB signalling proteins and/or ribosomal proteins.

We did not assess the functional implications of the altered EV proteomes after the FLT3 and/or Hh signalling inhibition. Among others, alterations in the EVs’ immunomodulatory capacities (Binder et al. 2021), their capabilities to transfer chemoresistance (Ma et al. 2014; Faict et al. 2019; Saadatpour et al. 2016; Asare‐Werehene et al. 2020; Feng et al. 2020; Wang et al. 2019) and their general impact on the microenvironment (Jella et al. 2018; Wang et al. 2021; Reale et al. 2022) are of key interest for future studies. However, inferring from the data gathered on AML patient EVs in this study and the comparison of AML cell lines to non‐AML cell lines by (Kang et al. 2021), we would speculate about lowered pro‐tumorigenic capabilities of EVs secreted by Creno‐ and HPI‐1‐treated AML cells.

EV release is mostly, but not always, referred to be enhanced after induction of chemotherapy and the cargo released is mostly described to have tumorigenic effects in the recipient cells (Ab Razak et al. 2019), however, as this is dependent on cell type and duration of the treatment, further research is needed to elucidate their contribution to metastasis, resistance and relapse. Similarly, a comprehensive characterization of ErbB signalling in AML, both within leukemic cells and the bone marrow microenvironment, and the cross‐talk via EVs could uncover novel vulnerabilities and therapeutic opportunities. A promising avenue for overcoming chemoresistance and relapse, and for improving outcomes in AML and other cancers, might be found in combining Creno treatment, which has already shown promising therapeutic outcomes (Wang et al. 2024) with ErbB‐signalling targeting therapies. In future studies, it will be important to functionally characterize the role of high‐level ribosomal and ErbB cargo in EVs from AML cells during progression, therapeutic response and resistance development, which may open new avenues for efficient combination therapies with FLT3 and Hh pathway inhibitors.

## 5 Conclusion

In summary, we demonstrated that, in contrast to the cellular proteome, treatment with the FLT3 inhibitor Creno, the Hh pathway and BET bromodomain inhibitor HPI‐1, or a combination of both, resulted in a downregulation of EV‐associated ribosomal proteins compared to untreated controls. Additionally, we revealed that treatment with Creno or the combination of both drugs led to a reduction of proteins assigned to the ErbB signalling pathway and other pro‐proliferative pathways in the vesicular proteome. Despite EVs secreted after chemotherapy are mainly described to be oncogenic, our study also points to a potential antitumorigenic effect of EVs secreted after treatment with Creno, which will be interesting to address in follow‐up studies. Thus, Creno treatment may potentially have a therapeutic effect by reducing the EV‐mediated transport of proliferative signalling proteins to recipient cells and might therefore also distort the transfer of chemoresistance.

## Author Contributions

**Constantin Blöchl**: conceptualization, investigation, writing ‐ original draft, methodology, writing ‐ review and editing, formal analysis. **Gabriele Blümel**: software, visualization, writing ‐ original draft, data curation, investigation, methodology, writing ‐ review and editing. **Martin Wolf**: investigation, methodology, writing ‐ review and editing, methodology, conceptualization. **Christof Regl**: writing ‐ review and editing, investigation, software, methodology, data curation, conceptualization. **Heide‐Marie Binder**: methodology, writing ‐ review and editing, investigation. **Suzana Tesanovic**: investigation, methodology, writing ‐ review and editing, conceptualization. **Daniel Lankes**: writing ‐ review and editing, investigation, methodology. **Nicole Maeding**: investigation, methodology, writing ‐ review and editing. **Peter W. Krenn**: investigation, writing ‐ review and editing, methodology, formal analysis, funding acquisition. **Dirk Strunk**: supervision, writing ‐ review and editing, funding acquisition, conceptualization, methodology. **Fritz Aberger**: conceptualization, funding acquisition, writing ‐ review and editing, supervision. **Christian G. Huber**: supervision, writing ‐ review and editing, funding acquisition, conceptualization, methodology.

## Conflicts of Interest

The authors declare no conflicts of interest.

## Supporting information



Supplemental Table S1

Supplemental Table S2

Supplemental Figures S1‐S8

Supplemental Checklist

## Data Availability

The mass spectrometry proteomics data and the R code have been deposited in the PRIDE repository (http://www.ebi.ac.uk/pride) with the data accession code PXD061404.
