# Supplementary material for: Selective EV Protein Sorting and Pathway Perturbation in AML Upon Synergistic FLT3 and Hedgehog Pathway Inhibition
Source: J Extracell Vesicles. 2025 Sep 23;14(9):e70163. doi: 10.1002/jev2.70163 (PMC12455877; doi:10.1002/jev2.70163)
Supplement: Supplementary file 3 — Supplemental Figures S1‐S8 [file JEV2-14-e70163-s001.pdf]

Supplementary Figure 1

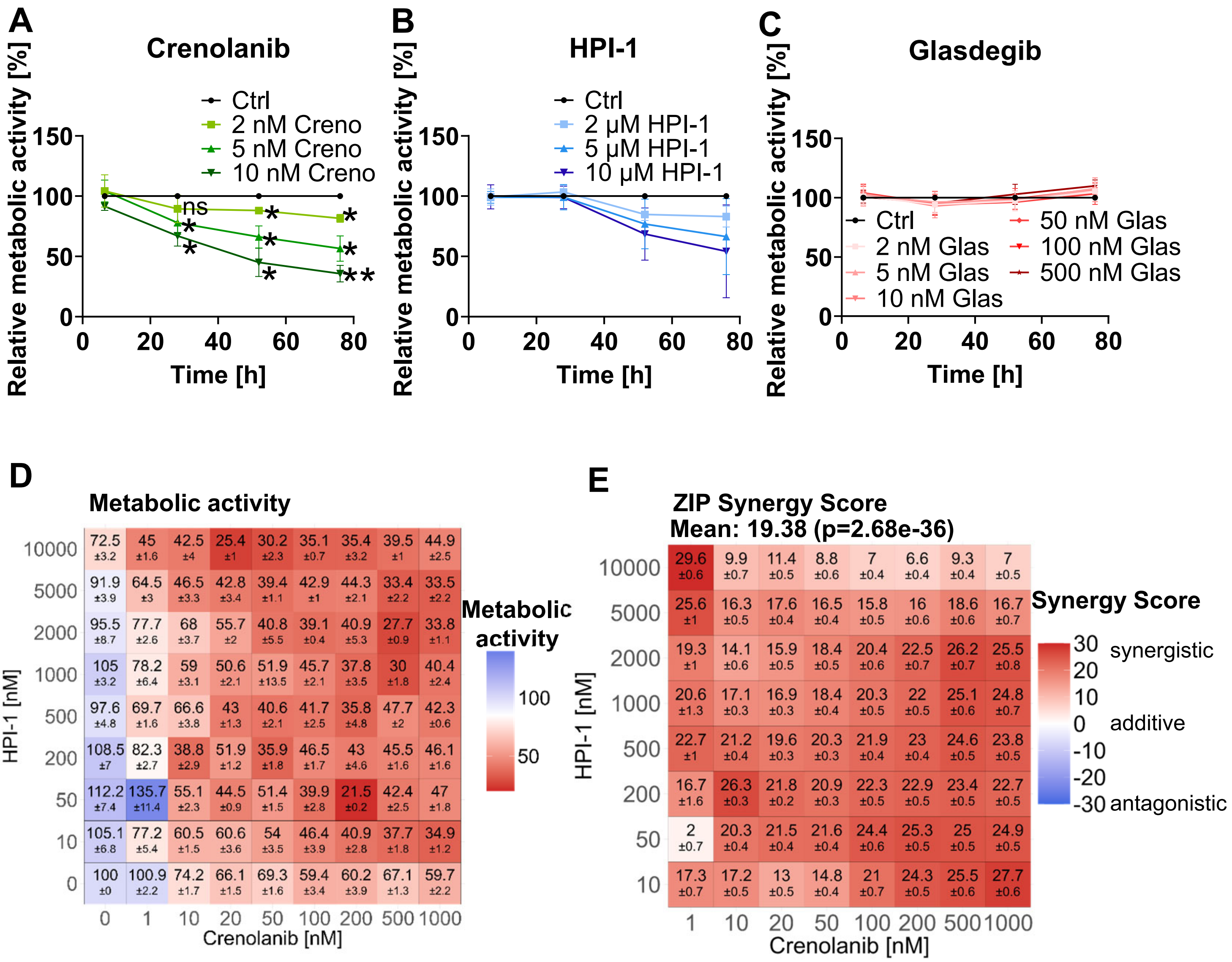

SUPPLEMENTARY FIGURE 1: Pharmacological inhibition of FLT3 and Hh signaling in the MV4-11 cell line. The effect of (A) Creno (B) HPI-1, (C) Glas on metabolic activity of MV4-11 cells was monitored during a time span of 78 h. Varying concentrations of (A) 2, 5, and 10 nM Creno, (B) 2, 5, 10 µM HPI-1 and (C) 2, 5, 10, 50, 100 and 500 nM Glas were tested compared to the 0.10% DMSO-treated control cells. All experiments are shown as biological triplicates, each consisting of three technical replicates. Differences were calculated with 2-way ANOVA, using Geisser Greenhouse correction and Dunetts Post hoc test: (D) and (E) An isobole analysis of Creno and HPI-1 treatment of MV4-11 cells was performed to determine synergistic and antagonistic effects (n=3). (D) The metabolic activity after 30 h in % compared to the control and additionally normalized to a treatment period of 7 h is depicted. Mean values and standard deviation are given. (E) Synergistic (red) and antagonistic (blue) effects are calculated according to the ZIP independence model based on the data presented in (D) (n=3) . *P < 0.05; **P < 0.01; n.s., not significant.

Supplementary Figure 2

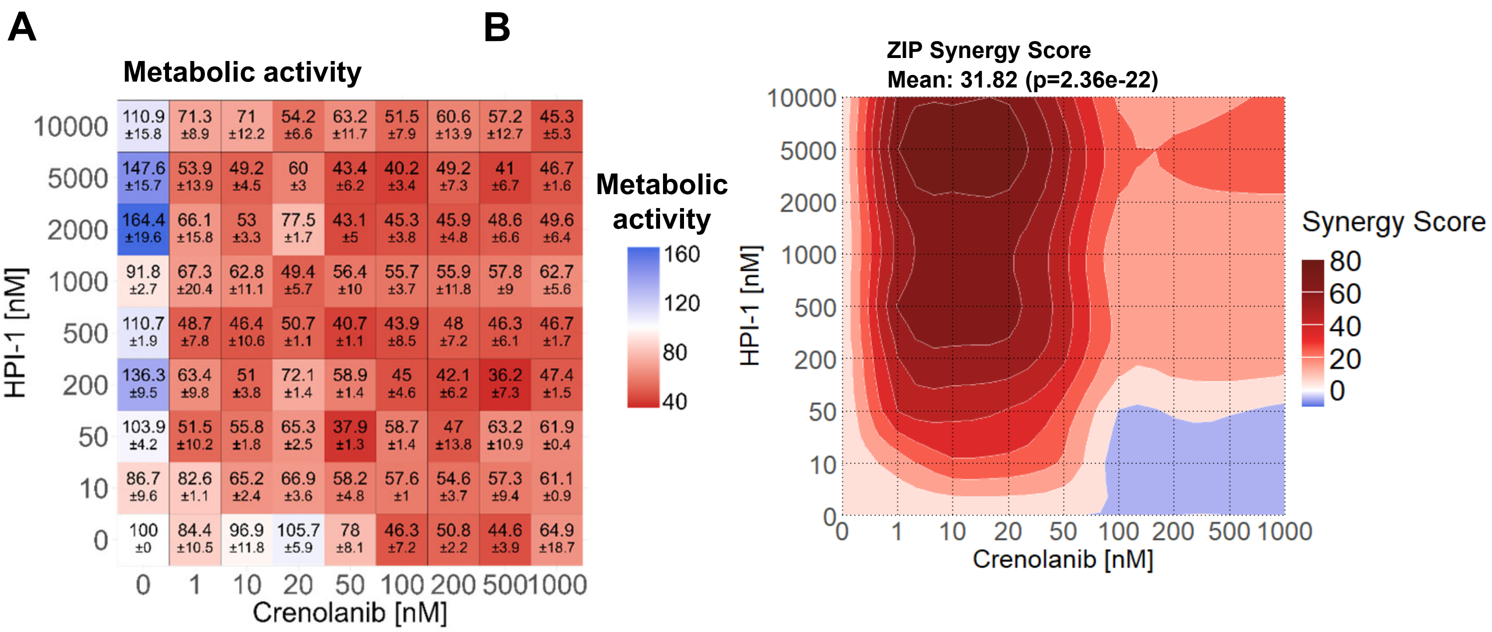

SUPPLEMENTARY FIGURE 2: Pharmacological inhibition of FLT3 and Hh signaling in the MOLM-14 cell line during cultivation in EV-depleted medium. (A) The metabolic activity after 30 h in % compared to the 0.10% DMSO-treated control cells and additionally normalized to a <u>treatment period of 7 h is depicted</u>. Mean values and standard deviation are given. (B) Synergistic (red) and antagonistic (blue) effects are calculated according to the ZIP independence model based on the data presented in (A) (n=2) .

Supplementary Figure 3

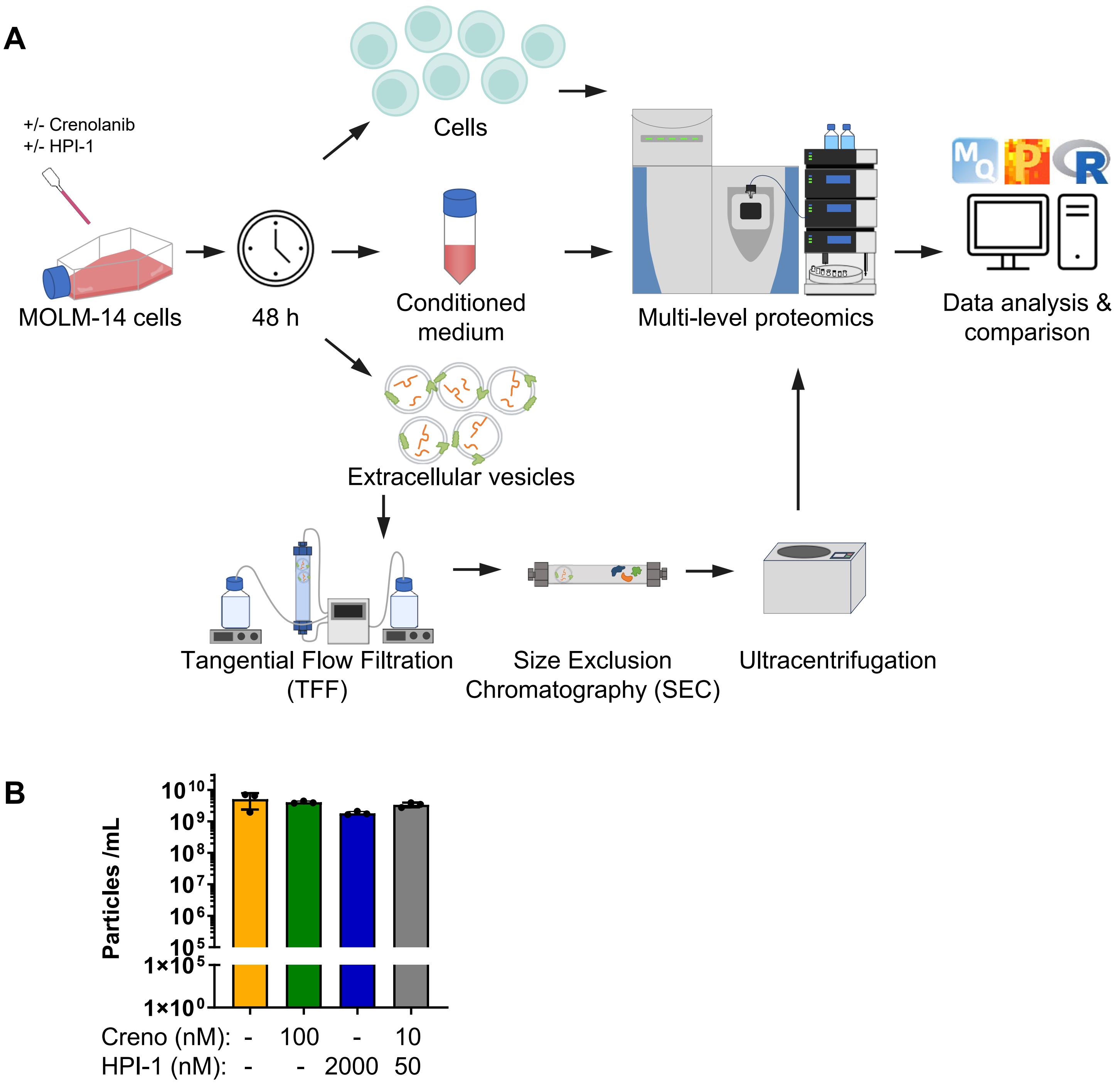

SUPPLEMENTARY FIGURE 3: Workflow for multi-level proteomics of the cells, the EVs and the conditioned medium and the EV purification and Tunable resistive pulse sensing (TRPS). (A) MOLM-14 cells were treated for 48 h with tangential flow filtered (TFF) media either containing 0.10% DMSO as a control or Creno and/or HPI-1. The cellular proteome and the proteome of the conditioned media were measured directly. EVs were pre-concentrated and purified from conditioned media by means of TFF and subsequent loading on a SEC column. For proteomics experiments, EVs were pelleted by ultracentrifugation and the supernatant was removed. Data was analyzed by MaxQuant, Perseus and R. (B) TRPS of EVs after fractionation with SEC. Obtained SEC fractions were characterized by TRPS (size and number), and for their protein content before fractions 2 and 3 were pooled. Particles/mL for each sample consisting out of biological triplicates are displayed as mean ± standard deviation.

Supplementary Figure 4

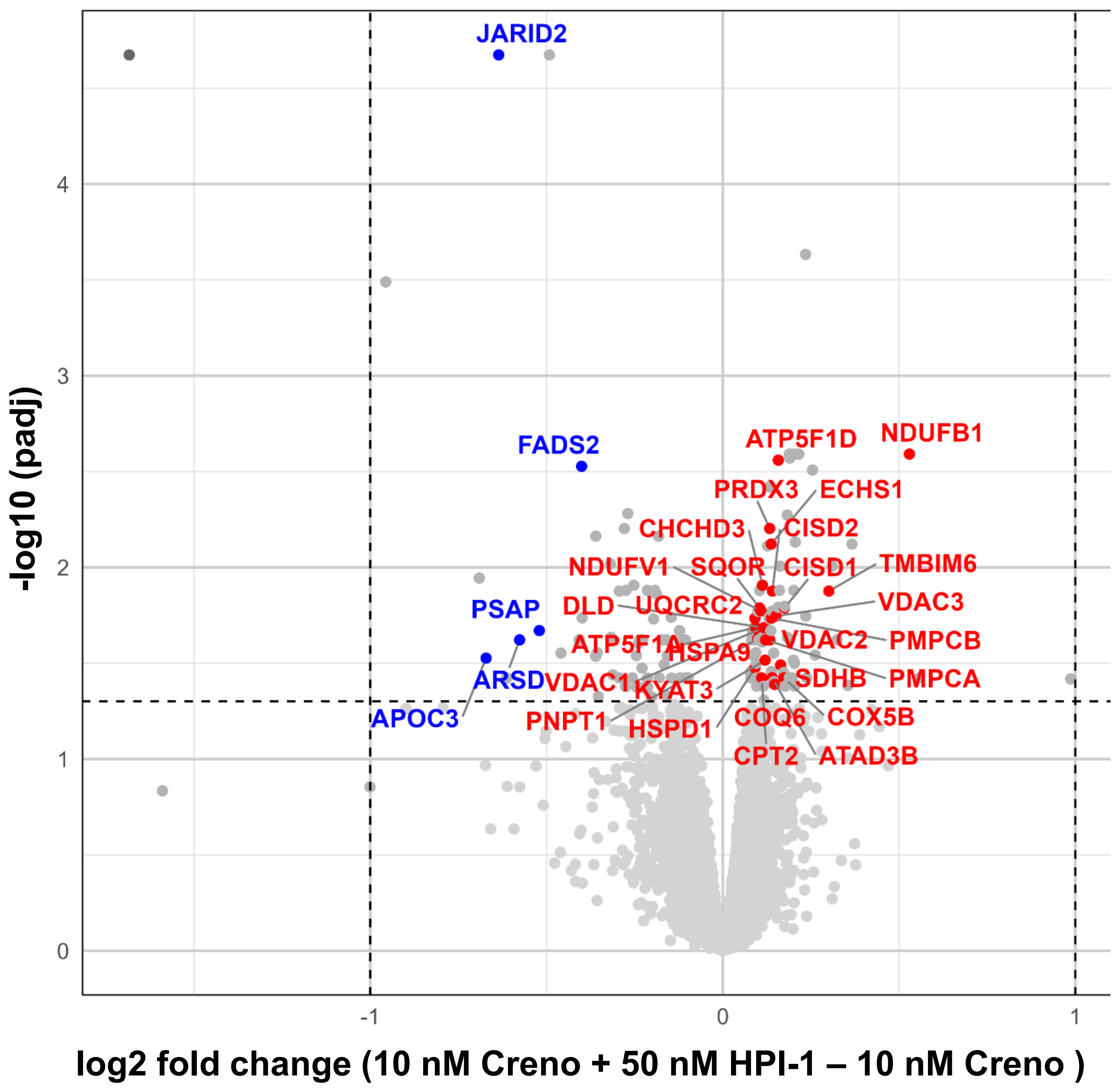

SUPPLEMENTARY FIGURE 4: Volcano plot depicting the alterations of the cellular proteomes of MOLM-14 cells treated with either 10 nM Creno or a combination of 10 nM Creno and 50 nM HPI-1. Significantly altered proteins associated with the lipid metabolism are marked in blue and those associated with oxidative phosphorylation in red.

Supplementary Figure 5

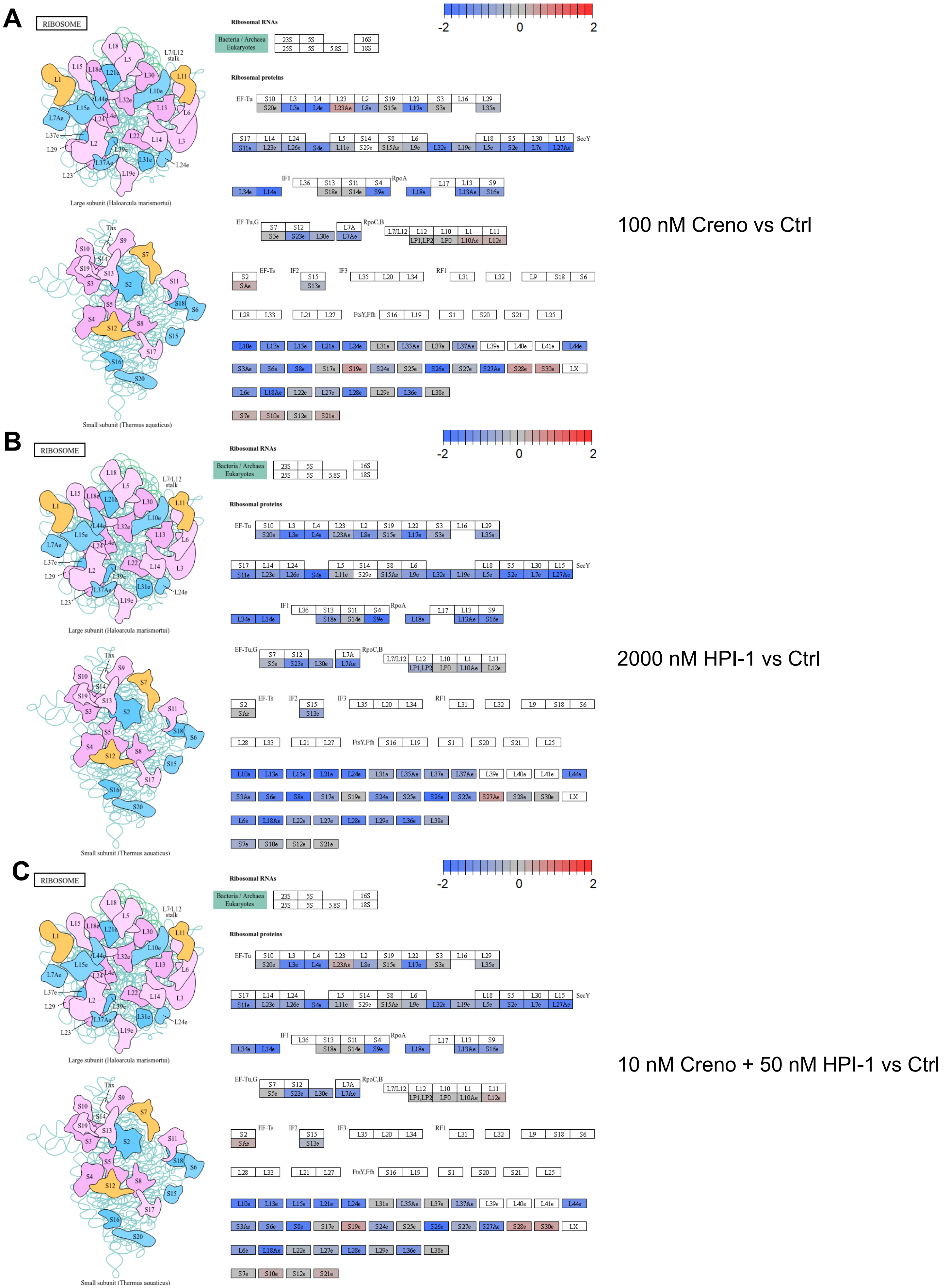

SUPPLEMENTARY FIGURE 5: Ribosomal proteins are highly downregulated in the vesicular proteome after pathway inhibition. (A) (B) (C) KEGG pathway of the ribosome (hs03011) is colored based on the expression levels comparing EVs treated with (A) 100 nM Creno, (B) HPI-1 and (C) 10 nM Creno + 50 nM HPI-1 to the 0.10% DMSO-treated control, calculated with LIMMA. Downregulation is depicted in blue, upregulation in red.

Supplementary Figure 6

A 2000 nM HPI-1 vs Ctrl

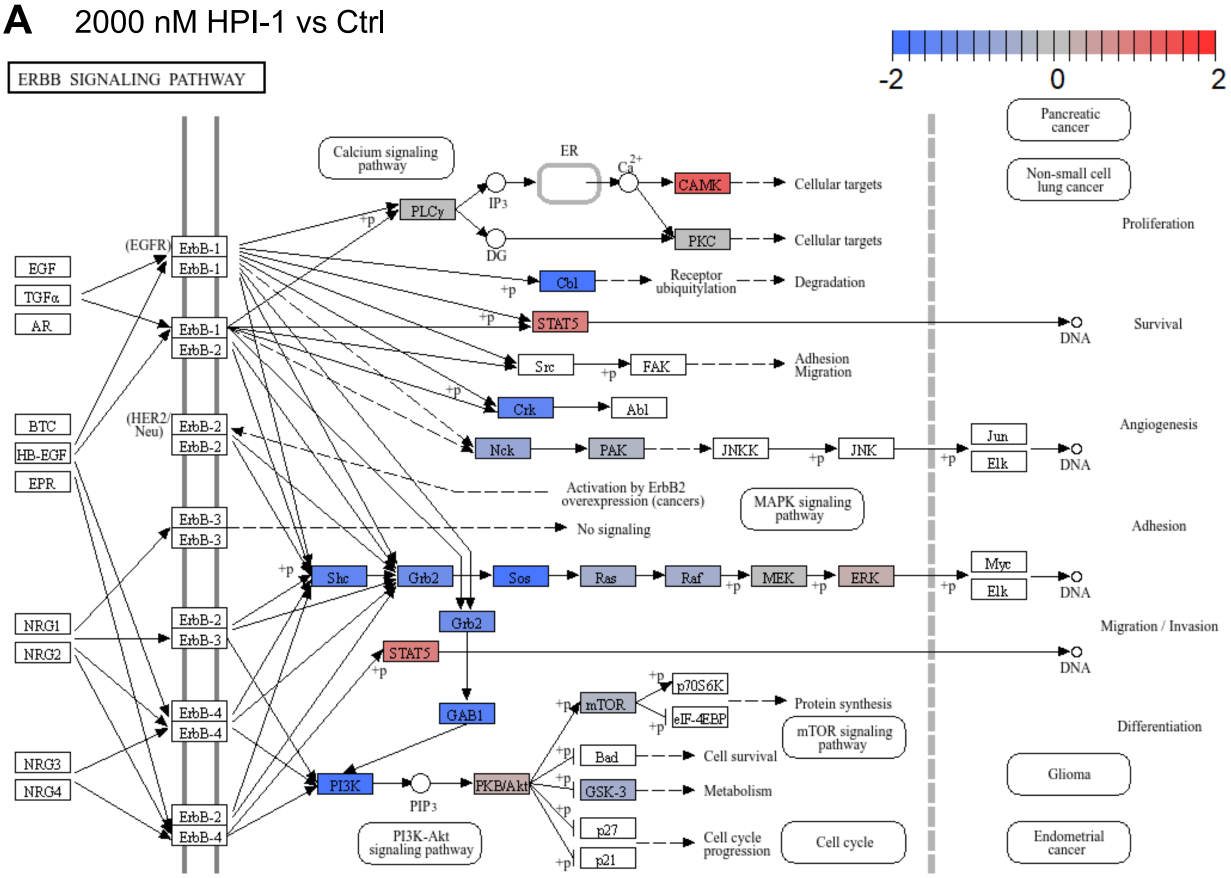

B 10 nM Creno + 50 nM HPI-1 vs Ctrl

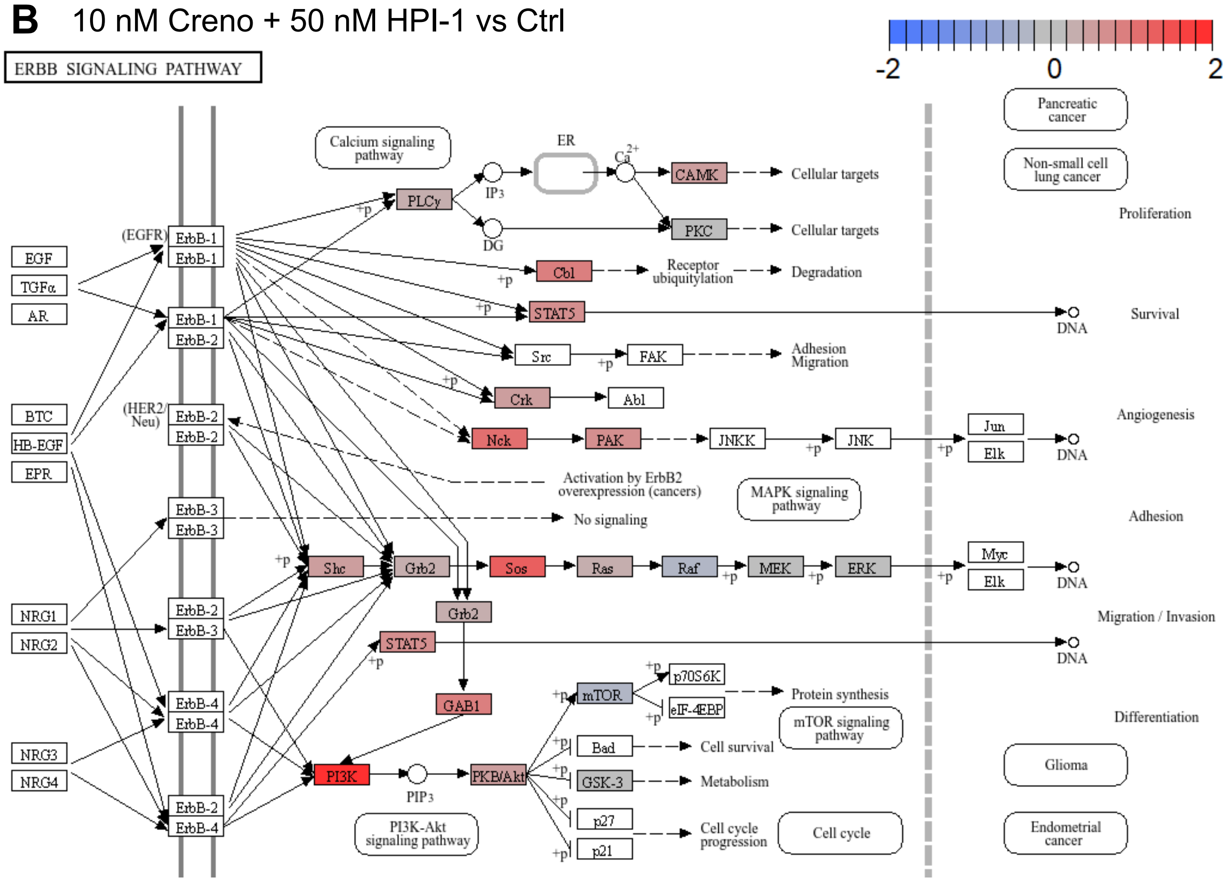

SUPPLEMENTARY FIGURE 6: Regulation of protein members of the ErbB signaling pathway in the vesicular proteome after pathway inhibition. (A) (B) The KEGG pathway of the ErbB signaling pathway (hs04012) is colored based on the expression levels comparing EVs treated with (A) HPI-1 and (B) 10 nM Creno + 50 nM HPI-1 to the 0.10% DMSO-treated control, calculated with LIMMA. Downregulation is depicted in blue, upregulation in red.

Supplementary Figure 7

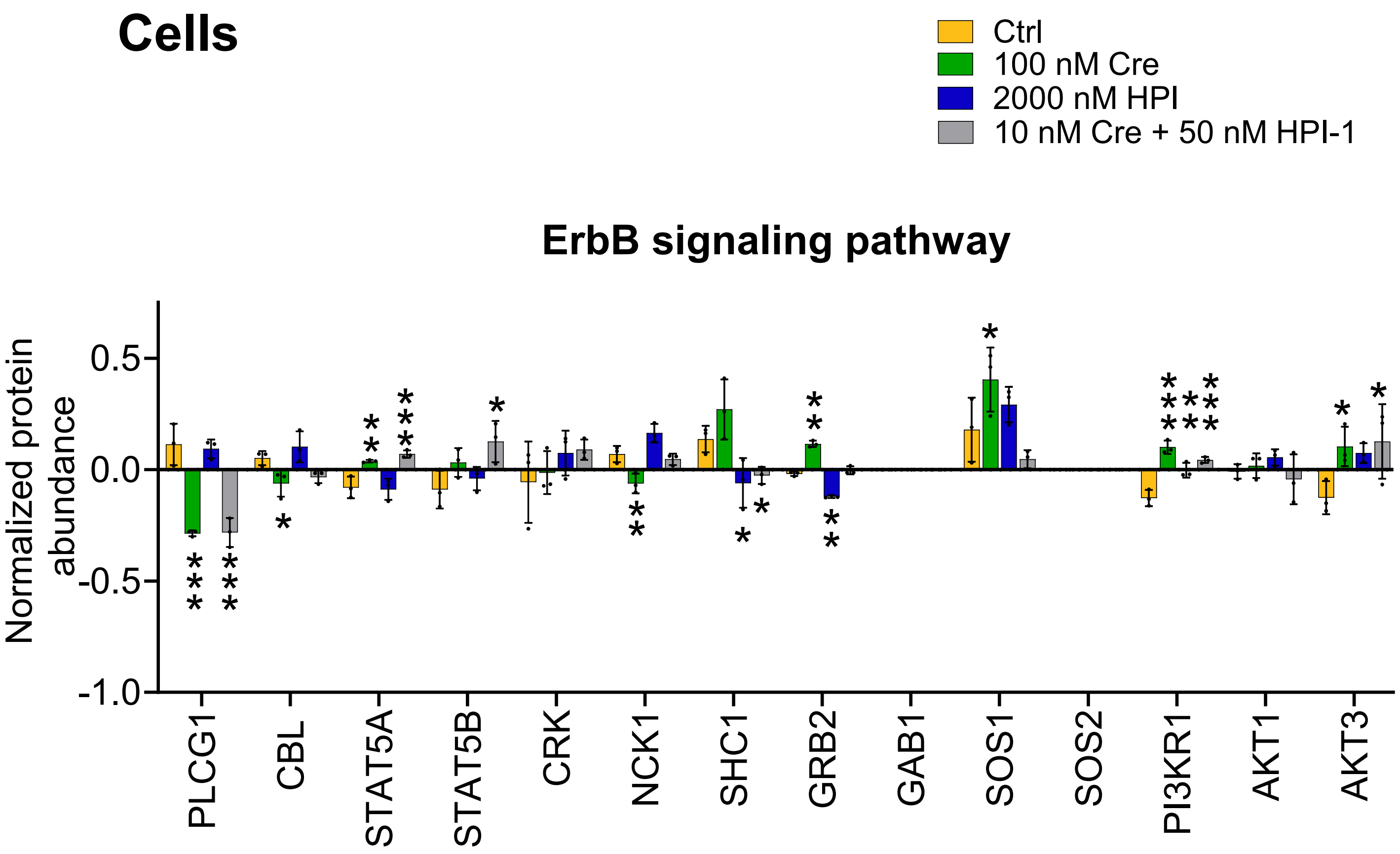

SUPPLEMENTARY FIGURE 7: Proteins involved in ErbB signaling are mostly unaltered in the cellular proteome. Cellular expression levels of starting proteins of sub-axis of the ErbB signaling pathway. Bars represent mean ± standard deviation of three biological replicates, differences were calculated with LIMMA. *P < 0.05; **P < 0.01; ***P < 0.001.

Supplementary Figure 8

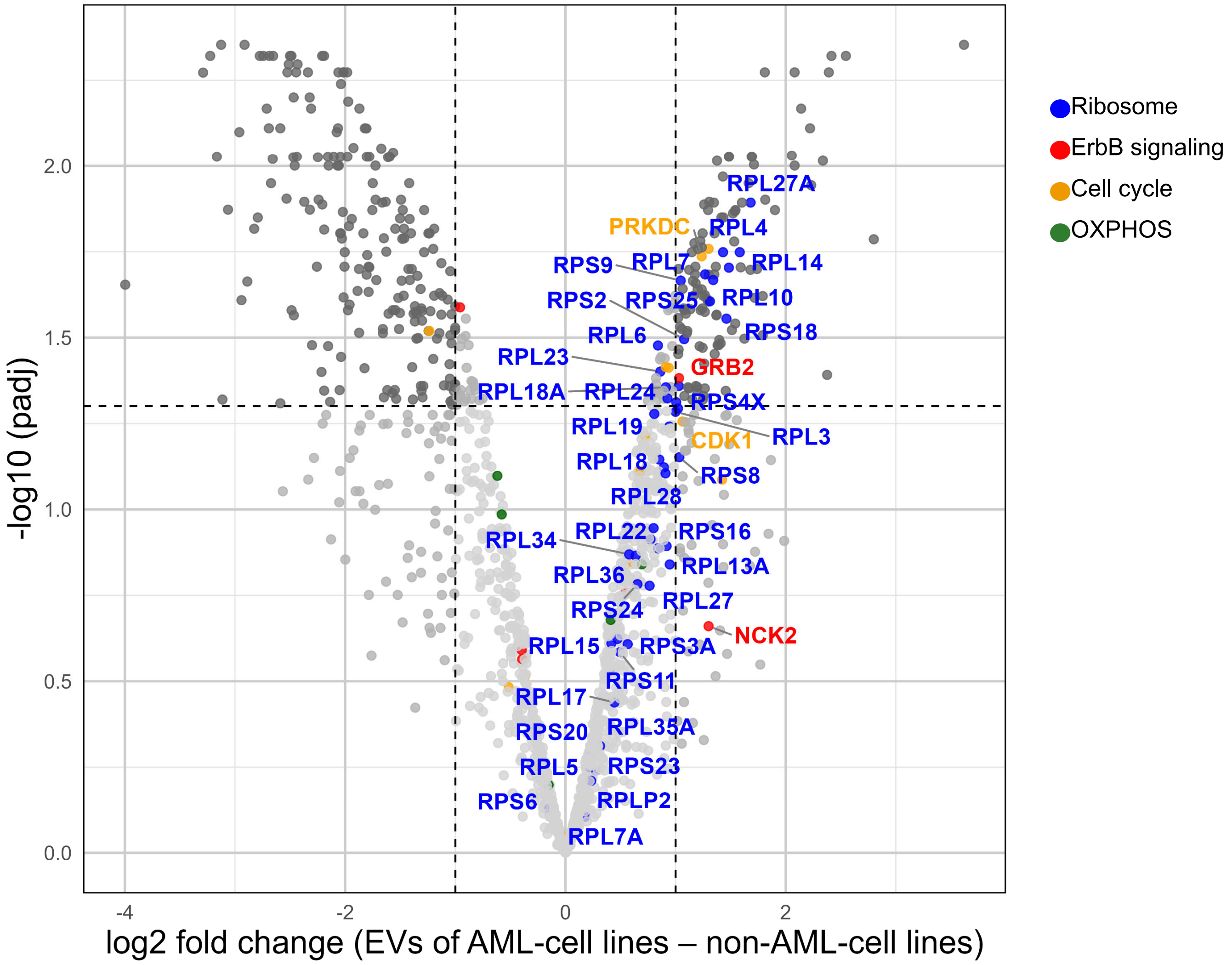

SUPPLEMENTARY FIGURE 8: Differential analysis of data from Supplemental Table S1 of Kang et al. 2021. Volcano plot depicting proteins altered in the EV proteome of three AML cell lines (KG-1, HL-60 and THP-1) and two non-AML cell lines (human dermofibroblasts (HDFa) and human mensenchymal stem cells (hMSCs)) calculated with LIMMA. The proteins are color-highlighted according to the respective KEGG pathway: Proteins associated to the Ribosome are colored in blue, proteins associated with ErbB signalling in red, proteins associated with Oxidative phosphorylation in green and proteins associated with Cell cycle in yellow. In addition, proteins were labeled with their protein name if they were identified in the EV proteome of MOLM-14 cells in the current study.

Kang, K. W.; Kim, H.; Hur, W.; Jung, J. H.; Jeong, S. J.; Shin, H.; Seo, D.; Jeong, H.; Choi, B.; Hong, S.; et al. A Proteomic Approach to Understand the Clinical Significance of Acute Myeloid Leukemia-Derived Extracellular Vesicles Reflecting Essential Characteristics of Leukemia. *Mol Cell Proteomics* **2021**, *20*, 100017. DOI: 10.1074/mcp.RA120.002169.
