# Supplementary material for: Selective EV Protein Sorting and Pathway Perturbation in AML Upon Synergistic FLT3 and Hedgehog Pathway Inhibition
Source: J Extracell Vesicles. 2025 Sep 23;14(9):e70163. doi: 10.1002/jev2.70163 (PMC12455877; doi:10.1002/jev2.70163)
Supplement: Supplementary file 4 — Supplemental Checklist [file JEV2-14-e70163-s003.pdf]

General information	
Title	Selective EV Protein Sorting and Pathway Perturbation in AML upon Synergistic FLT3 and Hedgehog Pathway Inhibition
Nomenclature used	Extracellular vesicles (EV)
Application	EV biology
EV-track ID	

Collection and storage		
Releasing cell information	Cell type origin	Animalia / / Blood (Plasma/Serum);Cell culture /
	Passaging	1
	Seeding density	75000 MOLM-14 cells/mL cells/cm²
	Cell viability	60-95%
	Culture volume	150 ml
	Culture vessel	T225
	Oxygen level	18% %
Culture conditions	Culturing medium	RPMI medium supplemented with 10% FBS and 1.0% L-alanyl-L-glutamine dipeptide
	Duration of cultivation	48h
	Harvesting medium	RPMI medium supplemented with 10% FBS particle depleted by TFF
	Cell count at harvest	2,5x10e5-1x10e6 cells
Storage and recovery	Condition medium	-80 °C
	EV preparations	-80 °C

EV isolation		
**Category**	**Methods used**	**Details**
Density based	Ultracentrifugation	EV fractions corresponding to 25 ug of protein were concentrated by ultracentrifugation (100,000 xg for 2.5 h)
Size based	size exclusion;tangential flow filtration	CM was concentrated about 100-fold by TFF employing a 300 kDa MWCO membrane (both Repligen) to a volume of approx. 1.5 mL.EV samples were further purified by means of size exclusion chromatography (SEC), using a qEV2 column (70nm, Izon Science Limited, Lyon, France)
Precipitation based	/	/
Affinity purification	/	/
Chromatography	/	/

EV isolation		
**Category**	**Methods used**	**Details**
Microfluidics	/	/

EV characterization				
		**Parameter**	**Unit**	**Method**
Quantification	Size & concentration	Particle number	1.8-5.2x10e9 particles/ml	RPS (TRPS/MRPS)
		Particle size	143 nm	RPS (TRPS/MRPS)
		Particle/protein ratio	particles/µg	
	Composition	Protein content	mg/ml	Bradford
		Lipid content	/	/
		RNA content	/	/
Identity		Category 1- Transmembrane or GPI-anchored proteins associated to plasma membrane and/or endosomes	CD63;CD81;CD9	/, Proteomics via Mass spec.
		Category 2- Cytosolic proteins recovered in EVs	ALIX;TSG101	/, Proteomics via Mass spec.
		Category 3- Major components of non-EV co-isolated structures	Albumin	/, Proteomics via Mass spec.
Visualization		/		/,

EV function(s)	
/	/

MiBlood-EV		
Blood study information	Time period of experiment (Years)	4
	Number of samples	9
	Biospecimen type	Plasma
	Biospecimen state	Frozen
	Source of frozen specimen	Obtained from biobank
	Patient fasting status	Uncertain
	Anatomical access site	venous blood
	Transport condition of tubes	Unknown
	Number of centrifugation cycles	2

MiBlood-EV		
Blood collection and processing	1st Centrifugation speed (RCF in x g)	450
	2nd Centrifugation speed (RCF in x g)	3000
	Additional processing steps (e.g. filtration)	For EV isolation, plasma samples were thawed, centrifuged at 2500x g for 15 min
	Storage temperature	-80°C
	Length of storage (range in years)	4
	Number of freeze-thaw cycles (range)	2
	Thawing temperature (°C)	RT
	Thawing duration (minutes)	1
Plasma/Serum quality control	Presence of hemolysis	Not tested
	Hemolized samples were discarded	No
	Presence of platelets	Not tested
	Western blot images provided in manuscript?	No

Other information
